# Twelve New Taxa of *Xylaria* Associated with Termite Nests and Soil from Northeast Thailand

**DOI:** 10.3390/biology10070575

**Published:** 2021-06-23

**Authors:** Niwana Wangsawat, Yu-Ming Ju, Cherdchai Phosri, Anthony J. S. Whalley, Nuttika Suwannasai

**Affiliations:** 1 Department of Biology, Faculty of Science, Srinakharinwirot University, 114 Sukhumvit 23, Wathana, Bangkok 10110, Thailand; niwana.wangsawat@g.swu.ac.th; 2 Institute of Plant and Microbial Biology, Academia Sinica, Nankang, Taipei 115, Taiwan; yumingju@gate.sinica.edu.tw; 3 Department of Biology, Faculty of Science, Nakhon Phanom University, Nakhon Phanom 48000, Thailand; Cherd.phosri@npu.ac.th; 4 School of Pharmacy and Biomolecular Science, Liverpool John Moore University, Liverpool L3 3AF, UK; A.J.Whalley@ljmu.ac.uk; 5 Department of Microbiology, Faculty of Science, Srinakharinwirot University, 114 Sukhumvit 23, Wathana, Bangkok 10110, Thailand

**Keywords:** *Pseudoxylaria*, Xylariaceae, Ascomycota, taxonomy, systematics

## Abstract

**Simple Summary:**

*Xylaria* species are known for their medicinal value and production of a variety of bioactive compounds. They usually grow on rotten wood, fallen leaves, seeds, and fruits. Some species can be found growing on soil or associated with termite nests, which belong to subgenus *Pseudoxylaria*. They share with other *Xylaria* species a suite of morphological characteristics, including conspicuous or massive upright stromata with a light-coloured interior, a longer than wide ascal apical ring, bluing in an iodine reagent, and brown unicellular ascospores possessing a germ slit. In Thailand, there are only limited reports on *Xylaria* diversity and taxonomy, especially on species associated with termite nests. In the present study, we describe 12 new *Xylaria* taxa and report two species closely resembling known species from termite nests or soil. Their morphological and cultural characteristics are described and illustrated, and their nucleotide sequences of ITS rDNA, alpha-actin, and beta-tubulin genes were obtained. Phylogenetic inference based on these sequences confirmed that all taxa analyzed belong to subgenus *Pseudoxylaria* and differ from all other species with sequences available in public databases. Our study is the first to report on the novel *Xylaria* species associated with termite nests or growing on soil in Thailand. Subgenus *Pseudoxylaria* is likely highly diverse in the country.

**Abstract:**

The diversity of *Xylaria* species associated with termite nests in northeast Thailand was investigated. Among the 14 taxa included in this study, 11 species and one variety were described as new, and another two species resemble the existing taxa, *X. escharoidea* and *X. nigripes*. The newly described taxa are *X. chaiyaphumensis*, *X. conica*, *X. fulvescens*, *X. ischnostroma*, *X. margaretae*, *X. minima*, *X. reinkingii* var. *microspora*, *X. siamensis*, *X. sihanonthii*, *X. subintraflava*, *X. thienhirunae*, and *X. vinacea*. Their morphological and cultural characteristics are described and illustrated, and their ITS, α-actin and β-tubulin sequences were analysed. A dichotomous key to the 17 species of *Xylaria* occurring in Thailand is provided.

## 1. Introduction

*Xylaria* Hill ex Schrank is the representative genus of the family Xylariaceae, being characterised by upright, cylindrical stromata with a light-coloured interior, eight-spored asci with an amyloid apical apparatus, brown unicellular ascospores possessing a germ slit, and a geniculosporium-like anamorph [1]. They are commonly found on fallen wood, leaves, fruits, seeds, dung, soil, and termite nests. These species associated with termite nests are mainly distributed in Africa and Asia, coinciding with the geographic range of the termite species belonging to the subfamily Macrotermitinae. Species associated with termite nests and growing on soil are classified in the subgenus *Pseudoxylaria* Boedijn [2]. Stromata of the species in subgenus *Pseudoxylaria* develop from abandoned fungus combs within termite nests and finally emerge on the soil surface [3]. The taxonomic characteristics of *Xylaria* species from termite nests and soil have long been discussed [3,4,5,6,7]. Recently, Hsieh et al. [8] described two new species, *X. insolita* and *X. subescharoidea*, from Taiwan, increasing the known species of subgenus *Pseudoxylaria* to 28.

In Thailand, there are only a few reports on *Xylaria* species of subgenus *Pseudoxylaria*. Thienhirun [9] studied the xylariaceous fungi throughout the country and reported only three species of *Xylaria* from termite nests: *X. gracillima*, *X*. cf. *melanaxis*, and *X*. cf. *nigripes*. Subsequently, Srihanant and Petcharat [10] surveyed the *Xylaria* species in oil palm and Pará rubber plantations in southern Thailand, and they found five species growing on soil that they identified as *X. acuminatilongissima*, *X. atrodivaricata*, *X. escharoidea*, *X. nigripes*, and *X. tanganyikaensis*. In the current study, we surveyed *Xylaria* species of subgenus *Pseudoxylaria* in northeast Thailand. Their morphological and culture characteristics were described, and their nucleotide sequences analysed to confirm their placement within subgenus *Pseudoxylaria*.

## 2. Materials and Methods

### 2.1. Sample Collection and Identification

Stromata of *Xylaria* species were collected from termite nests and soil in north-eastern Thailand during the rainy season (May–July) in the years 2015–2018. The morphological characteristics of stromatal surface and apex, perithecia, and ostioles were observed under a stereomicroscope (Carl Zeiss Stemi 508) and scanning electron microscope (SEM) (JEOL JSM-6610LVand JEOL JSM-IT500HR). The stromatal colour was recorded using Rayner’s colour chart [11]. Asci, ascospores, and conidia were mounted in water and Melzer’s iodine reagent for examination by bright-field microscopy (Olympus BX50) and differential interference contrast microscopy (Carl Zeiss Axio Imager A2). At least 40 ascospores and conidia per sample were examined. Cultures were obtained from the fresh inner tissue of stromata [6] or ascospores on potato dextrose agar (PDA) and oatmeal agar (OA). Cultures and anamorphs were observed after incubation at 30 °C for 7–21 days under 12 h fluorescent light. The studied specimens were deposited at Srinakharinwirot University Fungal Herbarium (SWUF), Bangkok, Thailand.

### 2.2. DNA Extraction, Amplification and Sequencing

Genomic DNAs were extracted from mycelia using a FavorPrep Plant Genomic DNA Extraction Mini Kit (Favorgen, Taiwan). The internal transcribed spacer region of nuclear rDNA (ITS) was amplified with the primer pair ITS5/ITS4 [12], beta-tubulin gene (TUB) was amplified with the primer pairs T1/T22 or T1/Bt2b, and Bt2a/T22 [13,14], while the alpha-actin gene (ACT) was amplified with the primer pair ACT-512F/ACT-738R [15]. Each PCR reaction consisted of 10–100 ng µL^−1^ of DNA template, 2 µM of each primer, 200 µM dNTP, 1.5 mM MgCl_2_, 1 × buffer, and 1 U µL^−1^ of Taq DNA polymerase (Qiagen, Germany). The PCR cycles contained an initial denaturation step at 94 °C for 5 min, 35 cycles of 94 °C for 1 min, 45–55 °C for 1 min, 72 °C for 1–1.30 min, and a final extension at 72 °C for 10 min [2,16]. The amplicons were directly sequenced or cloned using TOPclonerä TA kit (Enzynomics, Daejeon, Korea) following the manufacturer’s protocol. DNA sequencing was performed at the Apical Scientific Sdn. Bhd. company (Selangor, Malaysia) and the resulting chromatograms were manually checked using Chromas 2.4 (Technelysium Pty Ltd., South Brisbane, Australia).

### 2.3. ITS, Alpha-Actin and Beta-Tubulin Sequence Analysis

Two datasets were built, containing ACT-TUB and ITS sequences of *Xylaria* species associated with termite nest and related genera in the family Xylariaceae from GenBank, UNITE, and BOLD databases. They were aligned using MUSCLE software [17]. Several alignments of ACT-TUB sequences were performed merging or separating exon and intron regions. The final ACT-TUB datasets consisted of 1157 (exons + intons), 991 (exons only), and 165 (introns only) positions, while the ITS dataset had 747 positions. Gaps were treated as missing data. The phylogenetic trees were constructed using the maximum likelihood (ML) and Bayesian interference (BI) methods. ML trees were generated using the GTR+I+G model. The initial tree(s) for the heuristic search were obtained automatically by applying Neighbor-Join and BioNJ algorithms to a matrix of pairwise distances estimated using the maximum composite likelihood (MCL) approach and then selecting the topology with the superior log-likelihood value. The tree is drawn to scale, with branch lengths measured in the number of substitutions per site. The ML phylogenetic analysis was conducted in MEGA X [18,19]. The Bayesian inference analyses were performed with MrBayes v3.2 [20] by Markov chain Monte Carlo (MCMC) sampling. Six simultaneous Markov chains were run sampled every 100th generation. The analysis was stopped when the standard deviation of split frequencies between the trees generated in the two independent runs went below 0.01 after 1,000,000 generations. Twenty-five percent of the 10,000 sampled trees were discarded; the remaining were used to compute a 50% majority rule consensus tree to obtain estimates for posterior probabilities. The phylograms were visualised in FigTree 1.4.2 [21]. ITS sequences were analysed and used to confirm the *Xylaria* species identification carried out in this study. The trees were run at the same parameters as the ACT-TUB analysis. All sequences produced were deposited in the GenBank database.

## 3. Results

### 3.1. Phylogenetic Analyses

**Figure 1:**
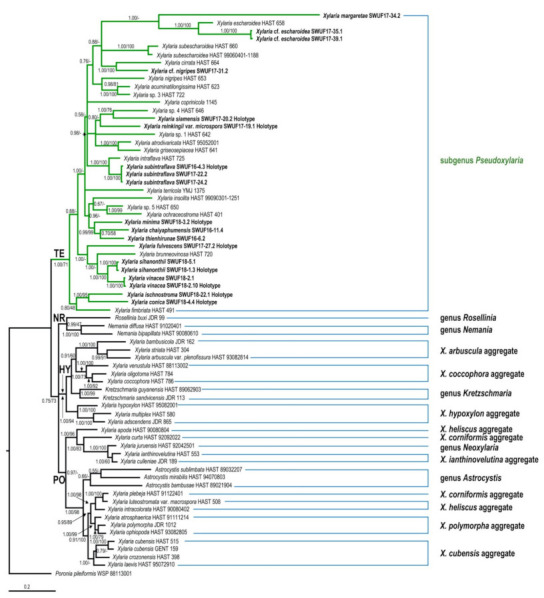
Phylogenetic tree inferred from Bayesian analysis based on ACT-TUB sequences of *Xylaria* and related genera. Branches are labelled with Bayesian posterior probabilities and bootstrap values from BI and ML analyses, respectively. *Poronia pileiformis* (WSP 88113001) is the outgroup.

**Figure 2:**
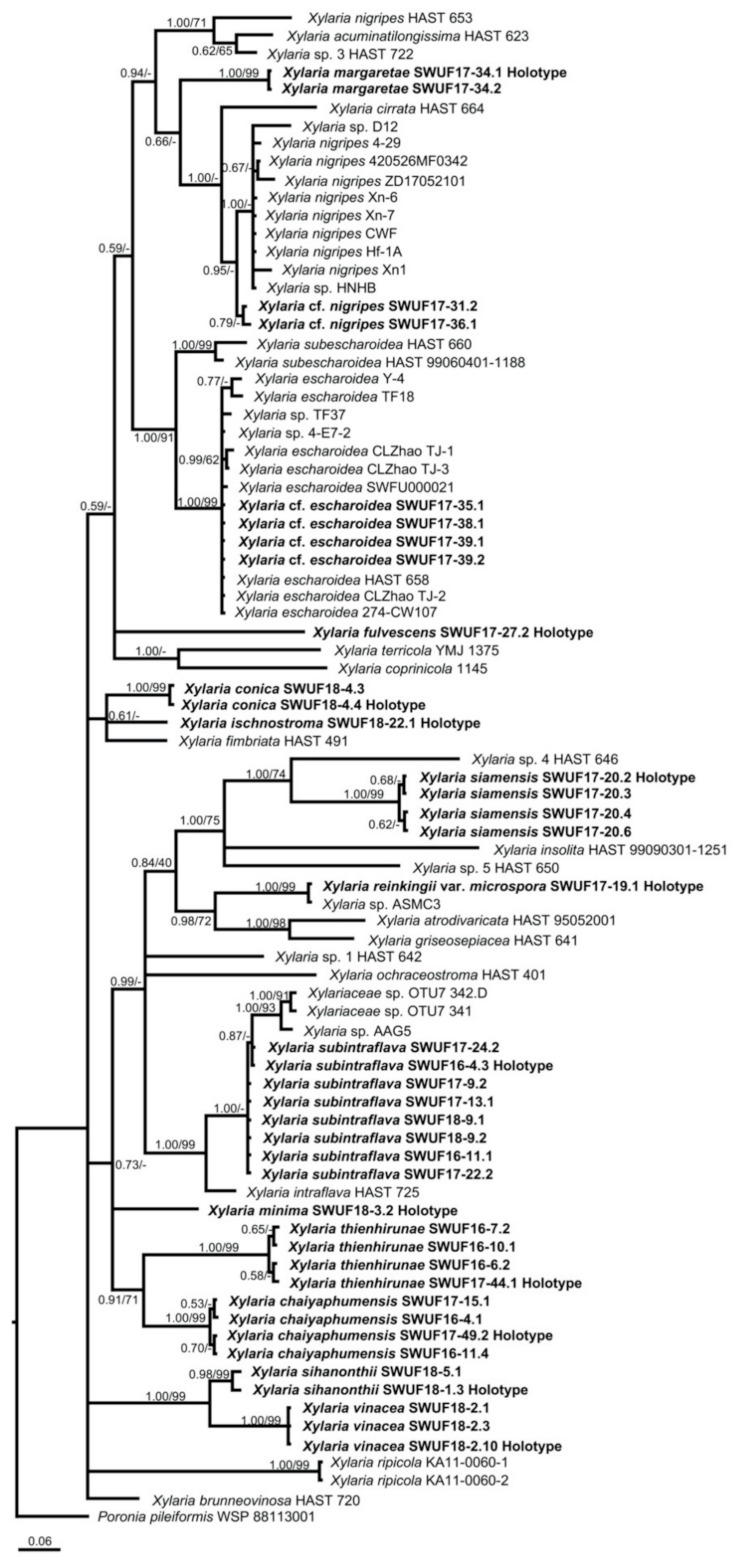
Phylogenetic tree inferred from Bayesian analysis based on a data set of ITS sequences of *Xylaria* and related species from termite nest or soil. Branches are labelled with Bayesian posterior probabilities and bootstrap values from BI and ML analyses, respectively. *Poronia pileiformis* (WSP 88113001) is the outgroup.

The GenBank accession numbers of ITS, ACT, and TUB sequences obtained in this study are listed in Table 1. The trees obtained from ACT-TUB datasets, including either exon only, intron only, or both regions, showed similar topologies in ML and BI. Therefore, the phylogenetic tree, including both exons and introns generated by BI analysis, was presented herein (Figure 1), while the tree generated by ML analysis is provided in supplementary data (Figure S1). The phylogenetic trees obtained from the exon-only and intron-only analyses are provided in Figures S2 and S3, respectively (supplementary data). All *Xylaria* taxa studied in the present work were grouped together with known species of *Xylaria* subgenus *Pseudoxylaria* in clade TE [2], with a high Bayesian posterior probability (1.00) and likelihood bootstrap value (71%). These taxa are clearly different from each other and from known species. Species of *Xylaria* subgenus *Pseudoxylaria* lacking sequences available in the public databases produce morphologically different teleomorphic and anamorphic states. Sequences of *Xylaria* species found on other substrates were grouped in clades HY and PO, while clade NR contained species of *Nemania* and *Rosellinia*, in accordance with the results obtained previously by Hsieh et al. [2]. In TE clade, *X. conica* (SWUF18-4.4) and *X. ischnostroma* (SWUF18-22.1), two species with larger ascospores (>10 µm in length), were grouped together with high bootstrap support with *X. fimbriata*, the only non-Asian species in this clade. *Xylaria subintraflava* (SWUF16-4.3, SWUF17-22.2, SWUF17-24.2) was grouped together with *X. intraflava*; their close relatedness agrees with their similar morphological characteristics. Similarly, *X. chaiyaphumensis* (SWUF16-11.4) and *X. thienhirunae* (SWUF16-6.2), which have a similar ascospore shape, colour, and size, were also significantly related on the basis of results from phylogenetic inference, based on 84 ITS sequences from *Xylaria* species associated with termite nests or soil from known species (Figure 2 and Figure S4 for ML analysis). Only *X. reinkingii* var. *microspora* (SWUF17-19.1) showed a 100% similarity to *Xylaria* sp. ASMC3 (GenBank accession number EU164404), obtained from a fungus comb of *Macrotermes carbonarius* in Vietnam [22]. Notably, ITS sequences of *X.* cf. *escharoidea* collected from Thailand showed a 99% similarity to *X. escharoidea* (herbarium voucher HAST 658, epitype; EU179864) [5], while ACT and TUB sequences obtained from the same voucher showed 99% and 97% similarities to the GenBank accession numbers GQ853044 and GQ502709, respectively. In the same way, *X.* cf. *nigripes* (SWUF17-31.1) sequences obtained in the present study showed 90.63%, 80.12%, and 81.78% similarities to the GenBank accession numbers GU324755 (ITS), GQ853045 (ACT), and GQ502710 (TUB) sequences of *X. nigripes* (herbarium voucher HAST 653), respectively, while its morphological characteristics were all similar to *X. nigripes* [5], with minor differences in perithecial size. Additional samples of *X.* cf. *escharoidea* and *X.* cf. *nigripes* from different localities are required to evaluate the variability of these species and understand their genetic boundaries.

### 3.2. Taxonomy

**Figure 3:**
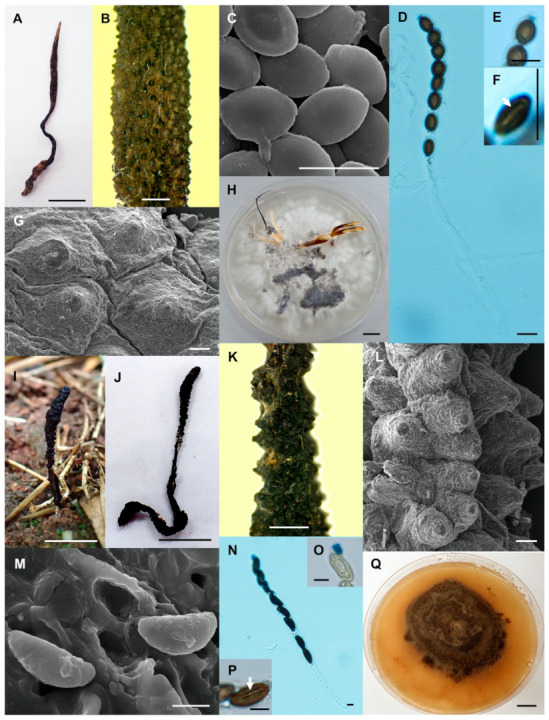
*Xylaria chaiyaphumensis* (SWUF17-49.2). (**A**) Stroma. (**B**) Wrinkled stromatal surface with ostioles. (**C**) Ascospores. (**D**) Ascus with ascospores. (**E**) Apical ring. (**F**) Germ slit (arrowed). (**G**) Wrinkled stromata surface with ostioles. (**H**) Colony on PDA in a 9 cm Petri dish at 4 weeks. *Xylaria conica* (SWUF18-4.4). (**I**) Natural habit of stroma. (**J**) Stromata. (**K**,**L**) Stromata surface with ostioles. (**M**) Ascospores. (**N**) Ascus with ascospores. (**O**) Apical ring. (**P**) Ascospore with germ slit (arrowed). (**Q**) Colony on PDA in a 9 cm Petri dish at 4 weeks. (**C**,**G**,**L**,**M**) by SEM; (**D**–**F**,**N**–**P**) by DIC. Scale bars (**A**,**H**–**J**,**Q**) = 1 cm; (**B**,**K**) = 1 mm; (**C**–**F**,**M**–**P**) = 5 µm; (**G**) = 20 µm; (**L**) = 100 µm.

1. *Xylaria chaiyaphumensis* Wangsawat N, Y.-M. Ju, Phosri C, Whalley AJS & Suwannasai N, sp. nov. Figure 3A–H.

MycoBank number: MB 839097.

Etymology—*Chaiyaphumensis* (Lat.): referring to Chaiyaphum Province of Thailand.

Type—Thailand, Dong Bang sub-district, Khon San District, Chaiyaphum province, on soil near termite nest, June 2017, Wangsawat N. SWUF17-49.2 (cultured) (holotype), GenBank accession: ITS = MT622775, α-act = MW459215; Dong Bang sub-district, Khon San District, Chaiyaphum province, on soil near termite nest, June 2017, Wangsawat N. SWUF17-4.1; Dong Klang sub-district, Khon San District, Chaiyaphum province, on termite nests, May 2016, Wangsawat N. SWUF16-04.1 (cultured), GenBank accession: ITS = MT622777; Thung Phra sub-district, Khon San District, Chaiyaphum province, on termite nests, May 2016, Wangsawat N. SWUF16-11.4 (cultured), GenBank accession: ITS = MT622776, α-act = MW459213, β-tub = MW459236; Thung Phra sub-district, Khon San District, Chaiyaphum province, on termite nests, June 2017, Khaeng-raeng R. SWUF17-15.1 (cultured), GenBank accession: ITS = MT622774, α-act = MW459214.

Stromata—Cylindrical, unbranched, with an acuminate apex, 5.9–7.6 cm in total length above ground by 2.5–4 mm broad, 1.7–2.6 cm long at fertile parts, 3.2–4.5 cm long at stipes, with a tortuose rooting base. The surface has conspicuous to inconspicuous perithecial mounds, longitudinally wrinkled, continuous, brown to blackish-brown, becoming light brown on the apex, black and smooth on the stipes, texture woody, interior white to buff (45). Perithecia globose to obovoid, 0.3–0.7 mm diameter by 0.6–0.7 mm high. Ostioles conic-papillate, black, ≤0.1 mm broad at base. Asci with eight ascospores, cylindrical, 52.5–128.5 µm total length by 4–5.5 µm broad, the spore-bearing parts 30–42(–45) µm long, stipes 20–91 µm long, with apical ring bluing in Melzer’s iodine reagent, inverted hat-shaped, 0.9–1.2 µm high by 0.9–1.5 µm broad.

Ascospores—Brown to dark brown, unicellular, lemon-shaped, nearly equilateral with narrowly rounded ends, sometimes pinched, smooth, 4–5(–5.7) × (2.5–)3–3.8 µm, with a straight germ slit 1/2 spore length.

Culture characteristics—Colonies reaching the edge of a 9 cm of Petri dish in 4 weeks, at first white and loose-cottony, becoming black surrounding the centre of colonies, azonate, mostly submerged, with diffuse margin. Reverse remaining uncoloured. Stromata cylindrical, tapering upward, unbranched to branched, flexuous, up to 9 cm long by 1–2 mm diameter, dispersed small stromata, white, becoming pale brown to black, white on the upper part. Anamorph not observed.

Notes—*Xylaria chaiyaphumensis* differs from the other species of *Xylaria* associated with termite nests in having lemon-shaped ascospores with a half spore-length germ slit. Stromata often exceed 3 mm diameter, and ascospores are mostly shorter than 5 µm.

2. *Xylaria conica* Wangsawat N, Y.-M. Ju, Phosri C, Whalley AJS & Suwannasai N, sp. nov. Figure 3I–Q.

MycoBank number: MB 839099.

Etymology—*Conica* (Lat.): referring to conical elevations.

Type—Thailand, Pang Ta Wai sub-district, Pang Sila Thong District, Kamphaeng Phet province, on termite nests, June 2018, Wangsawat N. SWUF18-4.4 (cultured) (holotype), GenBank accession: ITS = MT622787, α-act = MW459224, β-tub = MW459243; Pho Thong sub-district, Pang Sila Thong District, Kamphaeng Phet province, on termite nests, June 2018, Thamvithayakorn P. SWUF18-4.3 (cultured), GenBank accession: ITS = MT622786, α-act = MW459223; Thung Phra sub-district, Khon San District, Chaiyaphum province, on termite nest, May 2016, Wangsawat N. SWUF16-1.2.

Stromata—Cylindrical, unbranched with blunt apex, 3–5 cm in total length above ground by 1.5–2.5 mm broad, 0.9–2.4 cm long at fertile parts, 2.1–2.6 cm long at stipes, with a tortuose rooting base. The surface with mostly conspicuous to inconspicuous perithecial mounds, black, black and smooth stipes, texture woody, interior white. Perithecia obovoid to globose, 0.3–0.5 mm diameter by 0.3–0.9 mm high. Ostioles conic-papillate, black, 0.2–0.3 mm broad at base. Asci with eight spores, cylindrical, 150–168.5(–217.7) µm total length by 6.4–8.8 µm broad, the spore-bearing parts 73–110.5 µm long, stipes 59–79(–114) µm long, with apical ring bluing in Melzer’s iodine reagent, inverted hat-shaped, 4–4.5 µm high by 2–3 µm broad.

Ascospores—Brown to dark brown, unicellular, ellipsoid, inequilateral with narrowly rounded ends, smooth, (10–)10.8–12.3(–13.2) × 4.5–6(–6.4) µm, with a straight, spore-length germ slit on flattened side.

Culture characteristics—Colonies not reaching the edge of a 9 cm Petri dish in 8 weeks, attaining 4–6 cm diameter, at first white, cottony, immediately becoming dark brown, zonate, mostly submerged, with diffuse margin. Reverse pale brown. Stromata and anamorph not observed.

Note—This fungus differs from other *Xylaria* species associated with termite nests in having ascospores longer than 10 µm and larger perithecia. The perithecial mounds exposed from the stromatal surface have a conical shape. Although the ascospore size of *X. conica* is close to that of *X. radicans* (11.5–14 × 4–5 µm) [6], it has larger perithecia and a straight, spore-length germ slit. *Xylaria radicans* has perithecia 0.2–0.3 mm diameter and a straight germ slit shorter than spore length.

**Figure 4:**
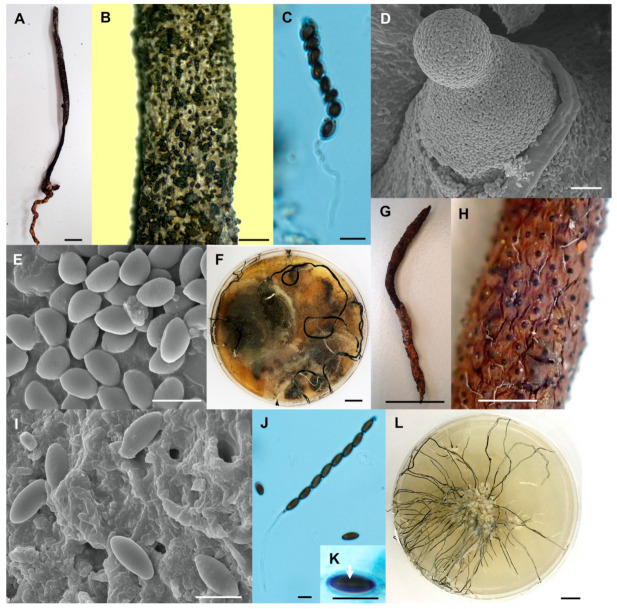
*Xylaria* cf. *escharoidea* (SWUF17–38.1). (**A**) Stroma. (**B**) Stromatal surface with ostioles. (**C**) Ascus with ascospores. (**D**) Ostiole. (**E**) Ascospores. (**F**) Colony on PDA in a 9 cm Petri dish at 4 weeks. *Xylaria fulvescens* (SWUF17-27.2). (**G**) Stroma. (**H**) Wrinkled stromatal surface with ostioles. (**I**) Ascospores. (**J**) Ascus and ascospores. (**K**) Germ slit (arrowed). (**L**) Colony on PDA in a 9 cm Petri dish at 4 weeks. (**D**,**E**,**I**) by SEM; (**C**,**J**,**K**) by DIC. Scale bars (**A**,**F**–**G**,**L**) = 1 cm; (**B**,**H**) = 1 mm; (**C**,**E**,**I**–**K**) = 5 µm; (**D**) = 20 µm.

3. *Xylaria* cf. *escharoidea* (Berk.) Fr. Figure 4A–F.

Type—Thailand, Dong Bang sub-district, Khon San District, Chaiyaphum province, on termite nests, June 2017, Wangsawat N. SWUF17-35.1 (cultured), GenBank accession: ITS = MT622792, α-act = MW459200, β-tub = MW459227; Thung Phra sub-district, Khon San District, Chaiyaphum province, on termite nests, June 2017, Phosri C. SWUF17-38.1 (cultured), GenBank accession: ITS = MT622793; Dong Klang sub-district, Khon San District, Chaiyaphum province, on termite nests, June 2017, Phosri C. SWUF17-39.1 (cultured), GenBank accession: ITS = MT622794, α-act = MW459201, β-tub = MW459228; Phosri C. SWUF17-39.2 (cultured), GenBank accession: ITS = MT622795.

The teleomorph is as described in Rogers et al. [5]. Apical rings are inverted hat-shaped, 1.4–1.7 µm high by 0.8–1.0 µm broad.

Culture characteristics–Colonies covering 9 cm Petri dish in 1 week, at first white, cottony-softly, becoming dark green to black, azonate, with diffuse margin. Reverse turning green-brown to dark green to black, due to release of exudate from mycelium. Stromata arise in 2–3 weeks, cylindrical, tapering upward, unbranched or branched, flexuous, up to 17 cm long by 1 mm diameter, white, becoming dark green to black. Anamorph not observed.

Note—This fungus differs from *X. escharoidea* [5] in having slightly larger perithecia, 0.3–0.5 mm diameter, and significantly different sequences of *β-tub* gene (97% similarity). Additional samples from different locations are required to clarify the identity of this species in future investigation.

4. *Xylaria fulvescens* Wangsawat N, Y.-M. Ju, Phosri C, Whalley AJS & Suwannasai N, sp. nov. Figure 4G–L.

MycoBank number: MB 839101.

Etymology—*Fulvescens* (Lat.): referring to the fulvous stromatal surface colour.

Type—Thailand, Thung Phra sub-district, Khon San District, Chaiyaphum province, on termite nests, June 2017, Wangsawat N. SWUF17-27.2 (cultured) (holotype), GenBank accession: ITS = MT622780, α-act = MW459218, β-tub = MW459238.

Stromata—Cylindrical, unbranched, with acuminate apex, 3.9 cm in total length above ground by 1.5–2.5 mm broad, 1–2.3 cm long at fertile parts, 1.6–2.8 cm long at stipes. Surface smooth with mostly embedded perithecia, wrinkled, brown to fulvous (43), black and smooth stipes, texture woody, interior white to cream. Perithecia subglobose to globose, 0.3–0.4 mm diameter by 0.4–0.5 mm high. Ostioles papillate, black, ≤0.1 mm broad at base. Asci with eight ascospores, cylindrical, 46.5–77(–88) µm total length by 3.4–5.0 µm broad, the spore-bearing parts 40–54 µm long, stipes 6.5–37 µm long, with apical ring bluing in Melzer’s iodine reagent, inverted hat-shaped, 1.1–1.7 µm high by 0.9–1.6 µm broad.

Ascospores—Light brown to dark brown, unicellular, ellipsoid, nearly equilateral with narrowly rounded ends, smooth, (4.5–)5–6.2 × 2.2–3 µm, with straight 3/4 spore length germ slit.

Culture characteristics—Colonies not reaching the edge of a 9 cm Petri dish in 4 weeks, attaining 3–4 cm diameter in 8 weeks, at first white, becoming pale brown, azonate, mostly submerged, with diffuse margin. Reverse remaining uncoloured. Stromata forming after 10 days, cylindrical, arising abundantly from the centre and elongating reach the edge of the plate, tapering upward, unbranched, flexuous, up to 10 cm long by <1 mm diameter, white, becoming smoke grey to black, white on the tips. Anamorph not observed.

Note—*Xylaria fulvescens* is similar to *X. ochraceostroma* [6] in stromatal surface and size but differs in having larger perithecia (0.3–0.4 mm diam), a smaller ascal apical apparatus (≤2 µm) and lighter stromatal surface colour. In addition, ascospores of *X. fulvescens* are ellipsoid and nearly equilateral, while those of *X. ochraceostroma* are ellipsoid and inequilateral.

**Figure 5:**
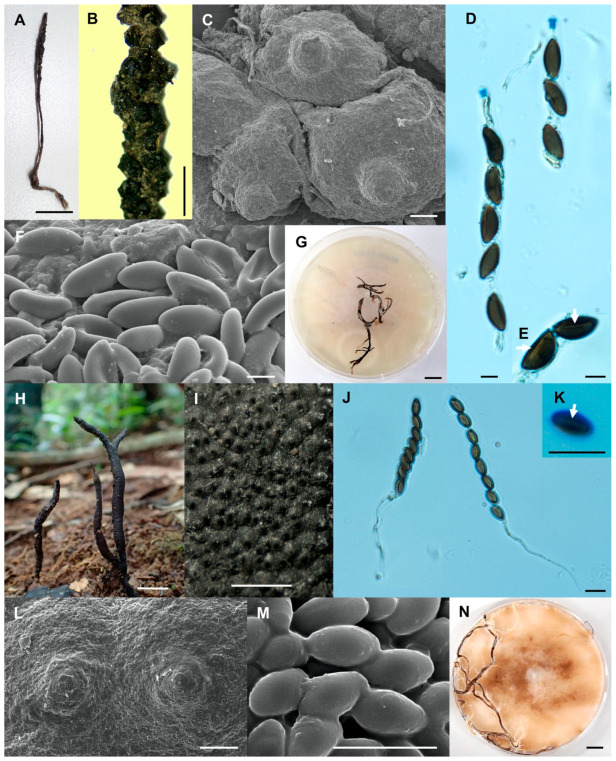
*Xylaria ischnostroma* (SWUF18-22.1). (**A**) Stroma. (**B**) Stromatal surface with ostioles. (**C**) Ostioles. (**D**) Ascospores and apical rings. (**E**) Ascospores with germ slits (arrowed). (**F**) Ascospores. (**G**) Colony on PDA in a 9 cm Petri dish at 4 weeks. *Xylaria margaretae* (SWUF17-34.1). (**H**) Natural habit of stromata. (**I**) Wrinkled stromata surface with ostioles. (**J**) Asci with ascospores. (**K**) Germ slit (arrowed). (**L**) Ostioles. (**M**) Ascospores. (**N**) Colony on PDA in a 9 cm Petri dish at 4 weeks. (**C**,**F**,**L**,**M**) by SEM; (**D**,**E**,**J**,**K**) by DIC. Scale bars (**A**,**G**,**H**,**N**) = 1 cm; (**B**,**I**) = 1 mm; (**C**,**L**) = 100 µm; (**D**–**F**,**J**,**K**,**M**) = 5 µm.

5. *Xylaria ischnostroma* Wangsawat N, Ju, Y.-M., Phosri C., Whalley A.J.S. & Suwannasai N., sp. nov. Figure 5A–G.

MycoBank number: MB 839102.

Etymology—*Ischnostroma* (Lat.): referring to its slim stromata.

Type—Thailand, Pho Thong sub-district, Pang Sila Thong District, Kamphaeng Phet Province, on termite nests, June 2018, Wangsawat N. SWUF18-22.1 (cultured) (holotype), GenBank accession: ITS = MT622788, α-act = MW459225, β-tub = MW459244.

Stromata—Cylindrical, unbranched, acuminate apices, 5.7 cm in total length above ground by 1–1.7 mm broad, 1.7 cm long at fertile parts, 4.0 cm long at stipes, with a tortuose rooting base. The surface with conspicuous perithecial mounds, of mostly naked perithecia, single to combined, blackish-brown, black and smooth stipes, texture woody, white to cream inner tissue. Perithecia globose, 0.5–0.6 mm diameter by 0.6–0.8 mm high. Ostioles conic-papillate, black, 0.1 mm broad at base. Asci with eight spores, cylindrical, 73–111 µm total length by 5.8–7.0 µm broad, with apical ring bluing in Melzer’s iodine reagent, inverted hat-shaped, 1.5–2 µm high by 2–3 µm broad.

Ascospores—Light brown to brown to dark brown, unicellular, ellipsoid, nearly inequilateral with narrowly rounded ends, some pinched at the ends, smooth, 10.3–11.6(–12.1) × 4.8–5.6 µm, with straight full spore length germ slit on flattened side.

Culture characteristics—Colonies covering a 9 cm Petri dish in 4 weeks, whitish, mostly submerged, azonate, with diffuse margin. Reverse pale brown. Stromata arising after 6 weeks or not produced, cylindrical, tapering upward, with several branches, up to 7.5 cm long by 1 mm diameter, at first white, becoming black. Anamorph not observed.

Note—*Xylaria ischnostroma* can be easily recognised because of its naked perithecial mounds and large ascospores. Although the stromatal features of this fungus are similar to those of *X. rhizomorpha* and *X. furcata* [5], these species have ascospores measuring 4.5–5 × 2–2.5 µm and 3.5–4.5(–5.5) × (2–)2.5–3 µm, respectively. The ascospore size of *X. ischnostroma* is closer to that of *X. radicans* [5] (11.5–14 × 4–5 µm), but their stromatal features are obviously different.

6. *Xylaria margaretae* Wangsawat N, Y.-M. Ju, Phosri C, Whalley AJS & Suwannasai N, sp. nov. Figure 5H–N.

MycoBank number: MB 839103.

Etymology—*Margaretae* (Lat.): referring to honour British mycologist Dr. Margaret Whalley, who had worked on Xylariaceae for more than 30 years.

Type—Thailand, Dong Klang sub-district, Khon San District, Chaiyaphum province, on termite nests, June 2017, Wangsawat N. SWUF17-34.1 (cultured) (holotype), GenBank accession: ITS = MT622778, α-act = MW459216; Thung Phra sub-district, Khon San District, Chaiyaphum province, on termite nests, June 2017, Wangsawat N. SWUF17-34.2 (cultured), GenBank accession: ITS = MT622779, α-act = MW459217, β-tub = MW459237.

Stromata—Cylindrical, unbranched or dichotomously branched once at tip, acuminate apices, 5.8–10 cm in total length above ground by 2–4 mm broad, 4–4.4 cm long at fertile parts, 1.4–6 cm long at stipes, with a tortuose rooting base sometimes. Surface with conspicuous to inconspicuous perithecial mounds, wrinkled, dark brick (60) to sepia (63) to fuscous black (104), black stipes, texture soft, interior buff (45). Perithecia obovoid to globose, 0.2–0.6 mm diameter by 0.3–0.5 mm high. Ostioles conic-papillate, black, 0.1–0.2 mm broad at base. Asci with eight ascospores, cylindrical, 66–99 µm total length by 3.4–5.2 µm broad, the spore-bearing parts 27–34 µm long, stipes 33–70 µm long, with apical rings straining blue in Melzer’s iodine reagent, inverted hat-shaped, 0.7–1.2 µm high by 0.7–1.2 µm broad.

Ascospores—Light brown to dark brown, unicellular, ellipsoid, inequilateral with narrowly rounded ends, smooth, (3.2–)3.8–5 × (1.8–)2–2.5 µm, with a full or nearly full spore length germ slit on convex side.

Culture characteristics—Colonies covering a 9 cm Petri dish in 1 week, at first white, cottony-softly, becoming red-brown, azonate, with diffuse margin. Reverse turning reddish-brown, due to release of exudate from mycelium. Stromata arise in 1–2 weeks or not produced, cylindrical, tapering upward, unbranched, sometimes dichotomously branched, flexuous, up to 13 cm long by 1 mm diameter, white, becoming reddish-brown. Anamorph not observed.

Note—*Xylaria margaretae* has unbranched or dichotomously branched stromata with acuminate apices similar to those of *X. atrodivaricata* [6], but stromata of *X. margaretae* are larger (>2 mm broad) with longer fertile parts. Perithecia of *X. atrodivaricata* are 0.2–0.3 mm in diameter by 0.3 mm high, while those of *X. margaretae* are 0.2–0.6 mm in diameter by 0.3–0.5 mm high. In addition, the ascal apical apparatus of *X. margaretae* is slightly smaller than that of *X. atrodivaricata*. In the present study, we found only one sample with dichotomously branched stromata. The ITS sequences of *X. atrodivaricata* and *X. margaretae* are only 89.53% similar, also supporting the idea that they are different species.

**Figure 6:**
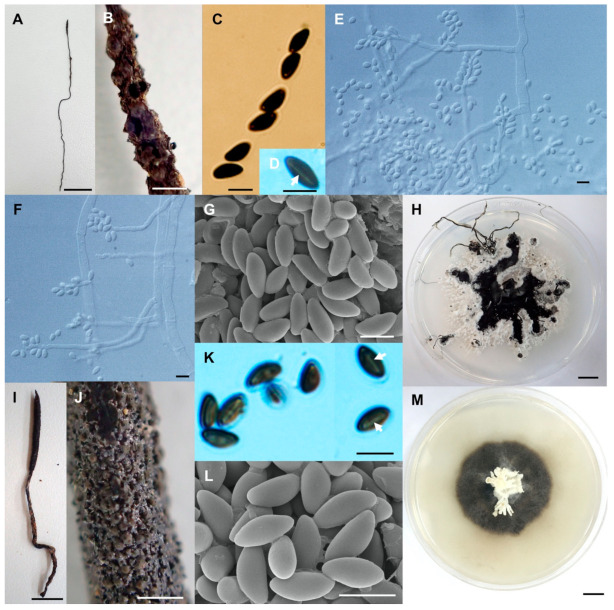
*Xylaria minima* (SWUF18-3.2). (**A**) Stroma. (**B**) Stromatal surface with ostioles. (**C**) Ascospores and apical ring. (**D**) Germ slit (arrowed). (**E**,**F**) Anamorph. (**G**) Ascospores. (**H**) Colony on PDA in a 9 cm Petri dish at 4 weeks. *Xylaria* cf. *nigripes* (SWUF17–31.2). (**I**) Stroma. (**J**) Stromata surface with ostioles. (**K**) Ascospores with germ slits (arrowed). (**L**) Ascospores. (**M**) Colony on PDA in a 9 cm Petri dish at 4 weeks. (**G**,**L**) by SEM; (**D**,**E**,**F**,**K**) by DIC. Scale bars (**A**,**H**,**I**,**M**) = 1 cm; (**B**,**J**) = 1 mm; (**C**,**D**,**E**–**G**,**K**,**L**) = 5 µm.

7. *Xylaria minima* Wangsawat N, Y.-M. Ju, Phosri C, Whalley AJS & Suwannasai N, sp. nov. Figure 6A–H.

MycoBank number: MB 839104.

Etymology—*Minima* (Lat.): referring to its tiny stromata.

Type—Thailand, Pho Thong sub-district, Pang Sila Thong District, Kamphaeng Phet province, on termite nests, June 2018, Wangsawat N. SWUF18-3.2 (cultured) (holotype), GenBank accession: ITS = MT622789, α-act = MW459226, β-tub = MW459245; Pho Thong sub-district, Pang Sila Thong District, Kamphaeng Phet province, on termite nests, June 2018, Khaeng-raeng R. SWUF18-3.1; Dong Klang sub-district, Khon San District, Chaiyaphum province, on termite nest, June 2017, Suwannasai N. SWUF17-1.1; Sakaerat Environmental Research Station Sakaerat Biosphere Reserves, Nakhon Ratchasima province, on termite nest, October 2018, Wangsawat N. SWUF18-2.4.

Stromata—Cylindrical, unbranched, acuminate apex, 4.7–7.5 cm in total length above ground by 0.5–1 mm broad, 0.5–1 cm long at fertile parts, 3.1–6.6 cm long at stipes. Surface with conspicuous perithecial mounds, hairy, longitudinally wrinkled, blackish-brown, white-cream on the apex, black and hairy stipes, texture soft, white to cream inner tissue. Perithecia globose, 0.2–0.4 mm diameter by 0.2–0.4 mm high. Ostioles slightly conic-papillate, black, 0.1 mm broad at base.

Asci with eight spores, cylindrical, 49.5–60 µm total length by 4.2–5 µm broad, the spore-bearing parts 36–43 µm long, stipes 13.5–17 µm long, with apical ring bluing in Melzer’s iodine reagent, inverted hat-shaped, 1.1–1.4 µm high by 1.4–2 µm broad.

Ascospores—Brown to dark brown, unicellular, ellipsoid-inequilateral, with narrowly rounded ends, smooth, 5.8–6.8 × 2.7–3.2 µm, with straight 3/4 spore length germ slit on flattened side.

Culture characteristics—Colonies on OA not reaching the edge of a 9 cm Petri dish in 4 weeks, at first white, cottony, becoming black, azonate, mostly submerged with diffuse margin. Reverse uncoloured. Immature stromata forming in 1 week, cylindrical, unbranched, up to 1.5 cm long by 1–2 mm diameter, white at first, becoming black-brown to black. White at tip due to conidia production. Anamorph—Conidiophores in upright, densely arranged palisades, some dichotomously branched from base, smooth, hyaline. Conidiogenous cells terminal, cylindrical, (20–)25–36(–48) × 1.8–3.5(–5.8) µm, smooth or slightly roughened, bearing several terminal and lateral denticulate conidial secession scars. Conidia produced holoblastically in sympodial sequence, hyaline, smooth, subglobose to obovoid, (3.2–)3.5–4.1(–4.7) × 2.1–2.9 µm, with a flattened base indicating former point of attachment to conidiogenous cell.

Note—*Xylaria minima* has solitary to confluent perithecial mounds similar to those of *X. ischnostroma*, but their ascospore sizes are different. *Xylaria minima* has smaller ascospores, resembling those of *X. piperiformis*, 5.5–7 × 3 × 3.5–4(–4.5) µm [5]. *Xylaria piperiformis* differs in having a cuboid ascal apical ring (1.5 µm) and a full-length germ slit on the flattened narrow side.

8. *Xylaria* cf. *nigripes* (Klotzsch) M. C. Cooke Figure 6I–M.

Specimens examined—Thailand, Thung Phra sub-district, Khon San District, Chaiyaphum province, on termite nests, June 2017, Wangsawat N. SWUF17-31.2 (cultured), GenBank accession: ITS = MT622790, α-act = MW459202, β-tub = MW459229; Thung Phra sub-district, Khon San District, Chaiyaphum province, on termite nests, June 2017, Phomphet N. SWUF17-36.1 (cultured), GenBank accession: ITS = MT622791, α-act = MW459203.

Stromata—Cylindrical, unbranched, acuminate and fertile apex, 6.4 cm in total length above ground by 2–5 mm broad, 2.4–3.9 cm long at fertile parts, 3.5–4 cm long at stipes, with a tortuose rooting base; surface with inconspicuous perithecial mounds, luteous (12), black stipes, woody and luteous interior layer. Perithecia obovoid, 0.2–0.4 mm diameter by 0.4–0.6(–0.8) mm high. Ostioles papillate, black, 0.1 mm broad at base. Asci absent. Ascospores light brown to dark brown, unicellular, ellipsoid, nearly equilateral with narrowly rounded ends, smooth, (4.17–)4.4–5.4 × 2.5–3.0 µm, with straight full spore length germ slit on flattened side.

Culture characteristics—Colonies covering a 9 cm Petri dish in 1 week, at first white, cottony-softly, becoming dark green to black, azonate, with diffuse margin. Reverse pale brown. Stromata arise in 2–3 weeks or not produced, cylindrical, tapering upward, unbranched or dichotomously branched, less than 2 cm long by 1–3 mm diameter, white to cream. Anamorph not observed.

Note—*Xylaria* cf. *nigripes* is quite similar to *X. nigripes* [5], due to its cylindrical fertile headed stromata, with a similar surface colour and hard texture. Ascospore shape and size are in the same range, (3.5–)4.5–5(–6) × 2–3 µm. However, this fungus differs from *X. nigripes* in having conspicuous perithecial mounds and slightly larger perithecia (0.2–0.4 mm diameter by 0.4–0.6(–0.8) mm high), while the perithecia of *X. nigripes* are more or less embedded and measure 0.1–0.2 mm diameter. In previous works, Thienhirun [9] identified some specimens from the Phetchaburi, Kanchanaburi, and Chanthaburi provinces in Thailand as *X*. cf. *nigripes*, but these had slightly smaller perithecia (0.2 mm diam) and smaller ascospores (3.8–4.3 × 1.9–2.5 µm) than our specimens.

**Figure 7:**
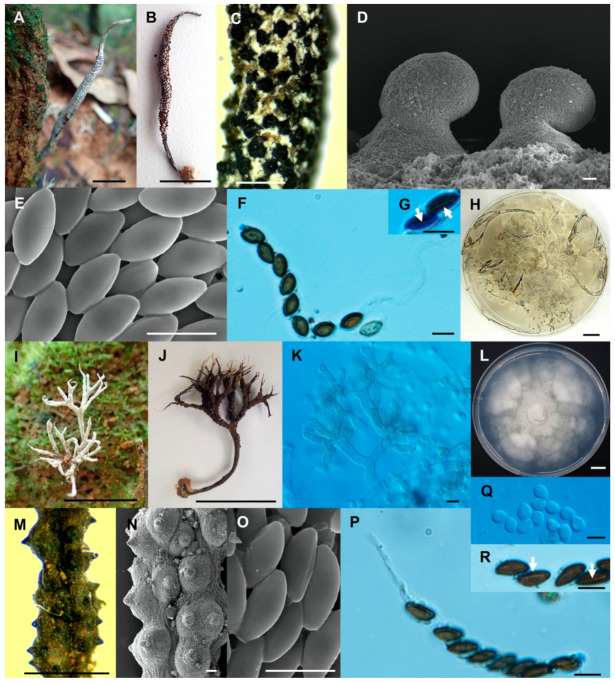
*Xylaria reinkingii* var. *microspora* (SWUF17-19.1). (**A**) Natural habit of stroma. (**B**) Stroma. (**C**) Stromatal surface with ostioles. (**D**) Ostioles with ascospores. (**E**) Ascospores. (**F**) Ascus with ascospores. (**G**) Germ slits (arrowed). (**H**) Colony on PDA in a 9 cm Petri dish at 4 weeks. *Xylaria siamensis* (SWUF17-20.3). (**I**) Natural habit of immature stromata. (**J**) Stroma. (**K**) Anamorph. (**L**) Colony on OA in a 9 cm Petri dish at 4 weeks. (**M**,**N**) Wrinkled stromata surface with ostioles. (**O**) Ascospores. (**P**) Ascus with ascospores. (**Q**) Conidia. (**R**) Ascospores and germ slits (arrowed). (**D**,**E**,**N**,**O**) by SEM; (**F**–**G**,**K**,**P**–**R**) by DIC. Scale bars (**A**,**B**,**H**–**J**,**L**) = 1 cm; (**C**,**M**) = 1 mm; (**D**) = 20 µm; (**E**–**G**,**K**,**O**–**R**) = 5 µm; (**N**) = 100 µm.

9. *Xylaria reinkingii* var. *microspora* Wangsawat N, Y.-M. Ju, Phosri C, Whalley AJS & Suwannasai N, var. nov. Figure 7A–H.

MycoBank number: MB 839105.

Etymology–*Microspora* (Lat.): a small-ascospored variety of *Xylaria reinkingii* C.G. Lloyd.

Type—Thailand, Thung Phra sub-district, Khon San District, Chaiyaphum province, on termite nests, June 2017, Wangsawat N. SWUF17-19.1 (cultured) (holotype), GenBank accession: ITS = MT622769, α-act = MW459209, β-tub = MW459234; Thung Phra sub-district, Khon San District, Chaiyaphum province, on termite nests, June 2017, Wangsawat N. SWUF17-19.2; Sakaerat Environmental Research Station Sakaerat Biosphere Reserves, Nakhon Ratchasima province, on termite nests, May 2013, Suwannasai N. SWUF13-1.

Stromata—Cylindrical, unbranched, with an acuminate apex, 2.2–12.3 cm in total length above ground, 1–4 mm broad, 0.9–5 cm long at fertile parts, 0.8–10 cm long at stipes, with a tortuose rooting base. The surface rough with mostly conspicuous perithecia mound, black, white powder outer layer, cracked into scales, black and smooth on the stipes, texture woody, interior buff (45), Perithecia globose, 0.2–0.5 mm diameter by 0.3–0.4 mm high. Ostioles conic-papillate, black, 0.1 mm diameter at base, 80–90 µm high from stroma. Asci with eight ascospores, cylindrical, 55–65 µm total length by 3.5–5 µm broad, the spore-bearing parts 34.5–45 µm long, stipes 13.5–27 µm long, with apical ring bluing in Melzer’s iodine reagent, inverted hat-shaped, 0.95–1.3(–1.67) µm high × (0.85–)1–1.5 µm broad.

Ascospores—Brown to dark brown, unicellular, ellipsoid, nearly equilateral to equilateral with narrowly rounded ends, some pinched at the ends, smooth, 5.4–6(–6.3) × 2.4–3.0 µm, with straight germ slit nearly full spore length on convex side.

Culture characteristics—Colonies reaching the edge of a 9 cm Petri dish in 4 weeks, at first white, softly and loose-cottony, becoming pale yellow, azonate, with diffuse margins. Reverse uncoloured. Stromata forming after 1 week, cylindrical, tapering upward, flexuous, unbranched or sometimes branched, up to 8 cm long by ≥1 mm diameter, black at base, becoming blackish or grey on surface, Smoke grey at tips. Anamorph not observed.

Note—This variety differs from *X. reinkingii* [6] in having smaller ascospores (≤6 µm) and a smaller apical apparatus of asci (≤2 µm).

10. *Xylaria siamensis* Wangsawat N, Y.-M. Ju, Phosri C, Whalley AJS & Suwannasai N, sp. nov. Figure 7I–R.

MycoBank number: MB 839096.

Etymology—*Siamensis* (Lat.): referring to Thailand.

Type—Thailand, Dong Klang sub-district, Khon San District, Chaiyaphum province, on termite nests, June 2017, Wangsawat N. SWUF17-20.2 (cultured) (holotype), GenBank accession: ITS = MT622765, α-act = MW459208, β-tub = MW459233; Dong Klang sub-district, Khon San District, Chaiyaphum province, on termite nests, June 2017, Wangsawat N. SWUF17-20.1; Dong Klang sub-district, Khon San District, Chaiyaphum province, on termite nests, June 2017, Wangsawat N. SWUF17-20.3, GenBank accession: ITS = MT622766; Dong Bang sub-district, Khon San District, Chaiyaphum province, on termite nests, June 2017, Phosri C. SWUF17-20.4, GenBank accession: ITS = MT622767; Dong Bang sub-district, Khon San District, Chaiyaphum province, on termite nests, June 2017, Phosri C. SWUF17-20.6, GenBank accession: ITS = MT622768.

Stromata—Cylindrical, highly branched with one or more tines, some dichotomously branched, bearing two acuminate apices, white at tip, 2–6.7 cm in total length above ground, 0.2–1.5 mm broad, 0.3–1 cm long at fertile parts, 1.3–5.3 cm long at stipes. Surface with conspicuous to half-exposed perithecial mounds, wrinkles, immature white, dark brick (60) to black when mature, overlain with some reddish-brown granules at base of stroma, interior white, black and smooth on the stipes. Texture woody to soft. Perithecia globose, 0.2–0.4 mm diameter by 0.2–0.3 mm high, crowded, highly conspicuous. Ostioles coarsely conic-papillate, black, ≤ 0.1 mm broad at base. Asci with eight ascospores, cylindrical, 45.4–74(–114.7) µm total length by 3.2–4.8 µm broad, the spore-bearing parts 30.5–42.5 µm long, stipes (5.4–)21–40.5(–83.8) µm long, with apical ring bluing in Melzer’s iodine reagent, inverted hat-shaped, 1.5–1.8 µm high × 1–1.5 µm broad. Paraphyses abundant.

Ascospore—Brown to dark brown, ellipsoid-inequilateral, with narrowly rounded ends, smooth, 5.0–6.0 × 2.5–3.5 µm, with a straight germ slit spore-length on flattened side.

Culture characteristics—Colonies on OA and PDA not reaching the edge of a 9 cm Petri dish in 4 weeks, at first white, becoming pale yellow, cottony in the centre, zonate, mostly submerged with diffuse margins. Reverse uncoloured. Stromata and anamorph not observed on culture media. Anamorph—Anamorphic characters observed on stromatal surface in nature. Conidiophores upright, dichotomously branched several times from base, smooth, hyaline, 3.5–5.3 µm at the main stipe. Conidiogenous cells terminal, flask-shaped, geniculate, 7.5–11.5 × 2.6–4.6 µm, smooth, bearing several terminal and lateral denticulate conidial secession scars. Conidia produced holoblastically in sympodial sequence, hyaline, smooth, obovoid, 4–4.6(–5) × 3–4 µm, with a flattened base indicating former point of attachment to conidiogenous cell.

Note—*Xylaria siamensis* is similar to *X. insolita* in having highly branched stromata and in the ascospore size ranges, but it mainly differs from *X. insolita* by a repeatedly dichotomous branching pattern and a dark brick to black stromatal surface colour without a greyish-brown outer layer ruptured by perithecial mounds. In addition, ascospores of *X. siamensis* are ellipsoid-inequilateral with narrowly rounded ends, while those of *X. insolita* have one end narrowly rounded and slightly beaked and the other end broadly rounded. The stromata of *X. siamensis* are similar to those of *X. furcata* and *X. furcata* var. *hirsuta* [5] in being highly dichotomously branched, but the stromata and the perithecia of *X. siamensis* are slightly smaller. The ascospores of *X. siamensis* are >5 µm, larger than those of *X. furcata*. Collection SWUF17-20.2 is finely pubescent between perithecia, but its ITS sequence is not different from that of the other collections.

**Figure 8:**
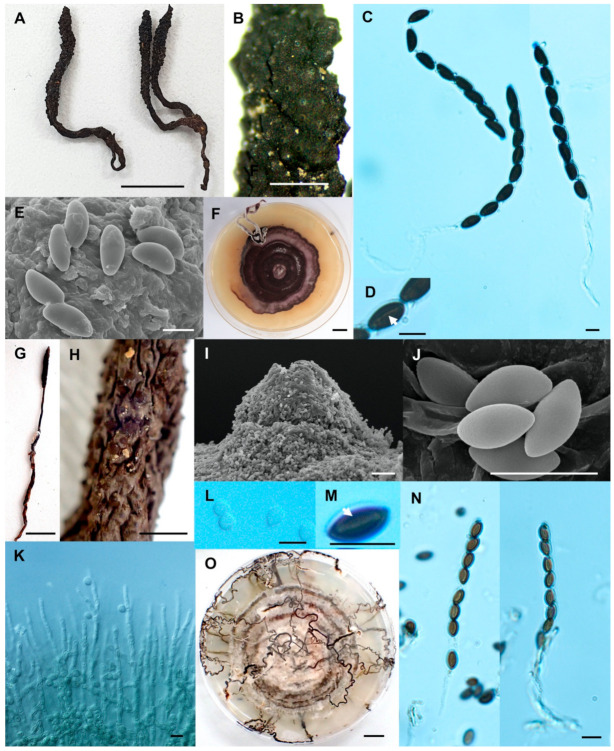
*Xylaria sihanonthii* (SWUF18-1.3). (**A**) Stromata. (**B**) Stromatal surface with ostioles. (**C**) Asci with ascospores. (**D**) germ slits (arrowed). (**E**) Ascospores. (**F**) Colony on PDA in a 9 cm Petri dish at 4 weeks. *Xylaria subintraflava* (PK17-24.2). (**G**) Stroma. (**H**) Wrinkled stromata surface with ostioles. (**I**) Ostiole. (**J**) Ascospores. (**K**) Anamorph. (**L**) Conidia. (**M**) Germ slit (arrowed). (**N**) Asci and ascospores. (**O**) Colony on PDA in a 9 cm Petri dish at 4 weeks. (**E**,**I**,**J**) by SEM; (**C**,**D**,**K**–**N**) by DIC. Scale bars (**A**,**F**,**G**,**O**) = 1 cm; (**B**,**H**) = 1 mm; (**C**–**E**,**J**–**N**) = 5 µm; (**I**) = 20 µm.

11. *Xylaria sihanonthii* Wangsawat N, Y.-M. Ju, Phosri C, Whalley AJS & Suwannasai N, sp. nov. Figure 8A–F.

MycoBank number: MB 839107.

Etymology—*Sihanonthii* (Lat.): referring to honour Thai mycologist Prof. Dr. Prakitsin Sihanonth, who had worked on fungi in Thailand for more than 30 years.

Type—Thailand, Nong Lat sub-district, Waritchaphum District, Sakon Nakhon province, on termite nests, July 2018, Wangsawat N. SWUF18-1.3 (cultured) (holotype), GenBank accession: ITS = MT622785, α-act = MW459222, β-tub = MW459242; Phang Khon sub-district, Phanh Khon District, Sakon Nakhon province, on termite nests, July 2018, Phosri C. SWUF18-5.1 (cultured), GenBank accession: ITS = MT622784, α-act = MW459221, β-tub = MW459241; Dong Bang sub-district, Khon San District, Chaiyaphum province, on termite nest, May 2016, Wangsawat N. SWUF16-29.1.

Stromata—Cylindrical, dichotomously branched one time at stipe, some unbranched with acuminate apices, 2.7–6.6 cm in total length above ground by 2–5 mm broad, 1–2.8 cm long at fertile parts, 2.1–3.8 cm long at stipes, some with two rooting bases. Surface with conspicuous perithecial mounds, blackish-brown to black, black and smooth stipes, texture woody, interior white. Perithecia obovoid, 0.3–0.6 mm diameter by 0.4–0.7 mm high. Ostioles conic-papillate, black, ≤0.1mm broad at base. Asci with eight spores, cylindrical, 77–120 µm total length by 4.4–6.1 µm broad, the spore-bearing parts 48–62(–70.5) µm long, stipes 19–49(–63) µm long, with apical ring bluing in Melzer’s iodine reagent, inverted hat-shaped, 1.2–1.7 µm high by 1.5–2.3 µm broad.

Ascospores—Light brown to dark brown, unicellular, ellipsoid, inequilateral with narrowly rounded ends, smooth, 7.5–9.5 × 3.5–4.5 µm, with straight full spore length germ slit on flattened side.

Culture characteristics—Colonies not reaching the edge of a 9 cm Petri dish in 8 weeks, at first white and loose-cottony, immediately becoming purple to brown vinaceous (84) zonate, with white concentric zones, mostly submerged, with diffuse margin. Reverse pale brown. Stromatal production beginning after 10 days, cylindrical, tapering upward, unbranched or branched, flexuous, up to 7 cm long by 1–2 mm diameter, black-purple, white at upper part, grey at top. Anamorph not observed.

Note—*Xylaria sihanonthii* has an ascospore size in the same range as *X. kedahae* (7.5–9 × 3–3.5 × 4–4.5 µm), *X. micrura* (7–8 × 3.5–4 µm) [6] and *X. brasiliensis* (7–8 × 3.5–4 µm) [5], but the shape of ascospores and stromata are different. *Xylaria kedahae* has ascospores with pinched ends. *Xylaria micrura* has a hyaline sheath surrounding ascospores. *Xylaria brasiliensis* has a gelatinous sheath surrounding ascospores and a less than spore-length germ slit.

12. *Xylaria subintraflava* Wangsawat N, Y.-M. Ju, Phosri C, Whalley AJS & Suwannasai N, sp. nov. Figure 8G–O.

MycoBank number: MB 839095.

Etymology—*Subintraflava* (Lat.): referring to its resemblance to *Xylaria intraflava*.

Type—Thailand, Dong Klang sub-district, Khon San District, Chaiyaphum province, on termite nests, May 2016, Wangsawat N. SWUF16-4.3 (cultured) (holotype), GenBank accession: ITS = MT622762, α-act = MW459204, β-tub = MW459230; Thung Phra sub-district, Khon San District, Chaiyaphum province, on termite nests, May 2016, Wangsawat N. SWUF16-11.1 (cultured), GenBank accession: ITS = MT622763, α-act = MW459205; Thung Phra sub-district, Khon San District, Chaiyaphum province, on termite nests, June 2017, Wangsawat N. SWUF17-9.2 (cultured), GenBank accession: ITS = MT622758; Dong Klang sub-district, Khon San District, Chaiyaphum province, on termite nests, June 2017, Wangsawat N. SWUF17-13.1 (cultured), GenBank accession: ITS = MT622759; Dong Klang sub-district, Khon San District, Chaiyaphum province, on termite nests, June 2017, Suwannasai N. SWUF17-22.2 (cultured), GenBank accession: ITS = MT622764, α-act = MW459206, β-tub = MW459231; Dong Klang sub-district, Khon San District, Chaiyaphum province, on termite nests, June 2017, Suwannasai N. SWUF17-24.2 (cultured), GenBank accession: ITS = MT622757, α-act = MW459207, β-tub = MW459232; Pang Ta Wai sub-district, Pang Sila Thong District, Kamphaeng Phet province, on termite nests, May 2018, Wangsawat N. SWUF18-9.1 (cultured), GenBank accession: ITS = MT622760; Pang Ta Wai sub-district, Pang Sila Thong District, Kamphaeng Phet province, on termite nests, May 2018, Wangsawat N. SWUF18-9.2 (cultured), GenBank accession: ITS = MT622761.

Stromata—Cylindrical, unbranched, with an acuminate apex, 2.1–7.9 cm long above ground by 1.5–3 mm broad, with fertile parts 0.85–1.7 cm long, 0.4–5.4 cm long at stipes, with a tortuose rooting base; surface smooth except roughened around ostioles, with slight perithecial mounds, longitudinally wrinkled, continuous, blackish-brown, fawn (87), dark brick (60), some with glossy on fertile part, dark brick (60) to black on smooth stipes, interior pale luteous (11). Perithecia obovoid to globose, 0.3–0.7 mm diameter by 0.3–0.8 mm high. Ostioles conic-papillate, black, ≤0.1 mm broad at base. Asci with eight ascospores, cylindrical, 40–70 µm total length by 4–5 µm broad, the spore-bearing parts 25–31 µm long, stipes 11–38 µm long, with apical ring bluing in Melzer’s iodine reagent, inverted hat-shaped, 0.8–1µm high × 0.8–1 µm broad. Paraphyses abundant.

Ascospores—Brown to dark brown, unicellular, ellipsoid, equilateral with narrowly rounded ends, smooth, 3.5–5 × 1.8–2.5 µm, with a straight germ slit 3/4 spore length.

Culture characteristics—Colonies on OA not reaching the edge of a 9 cm of Petri dish in 4 weeks, at first white, becoming brown patched, mostly submerged, zonate with diffuse margin. Reverse uncoloured. Immature stromata forming after 1 week, cylindrical, unbranched, up to 1 cm long by ≤0.1 mm diameter, black to grey at base, grey on surface. Smoke grey at tip and some on aerial mycelia due to production of conidia. Anamorph—Conidiophores in upright, densely arranged palisades, dichotomously branched several times from base, smooth, hyaline to light brown. Conidiogenous cells terminal, cylindrical, geniculate, 9.5–17(–25) × 1.6–2.4 µm, smooth or roughened, bearing several terminal and lateral denticulate conidial secession scars. Conidia produced holoblastically in sympodial sequence, hyaline to light brown, smooth, globose to subglobose 2.9–3.9 × 2.8–3.2 µm, with a flattened base indicating former point of attachment to conidiogenous cell.

Note—*Xylaria subintraflava* is similar to *X. intraflava* [6] in having slight perithecial mounds, a wrinkled stromatal surface, and small ascospores ≤5 µm long. It differs from *X. intraflava* by having unbranched stromata with one acuminate apex, larger perithecia up to 0.7 mm diameter, ascospores ellipsoid, nearly equilateral with narrowly rounded ends that are not pinched, and a small ascal apical apparatus less than 1 µm high. Furthermore, the inner stromatal tissue is pale luteous (11), while that of *X. intraflava* is pure yellow (14). The pairwise comparison of ITS sequences between *X. subintraflava* and *X. intraflava* revealed a 94.75% similarity.

**Figure 9:**
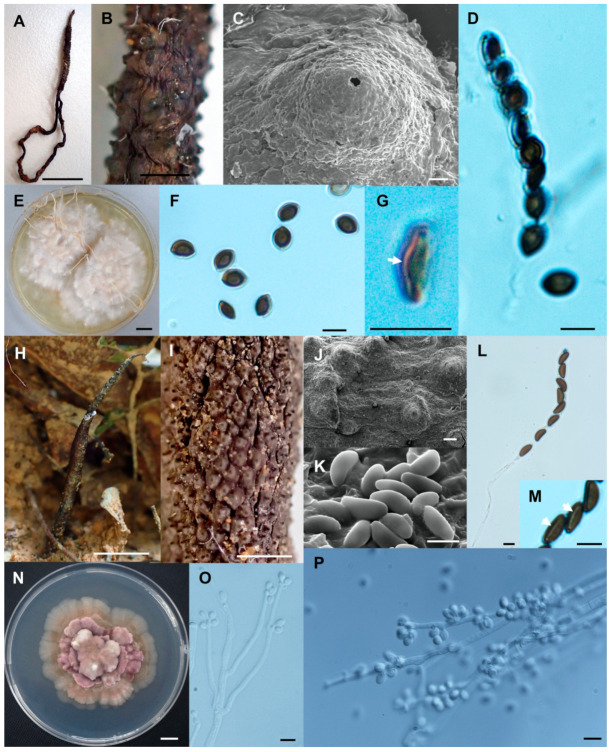
*Xylaria thienhirunae* (SWUF17-44.1). (**A**) Stroma. (**B**) Wrinkled stromatal surface with ostioles. (**C**) Ostiole. (**D**) Ascus and ascospores. (**E**) Colony on PDA in a 9 cm Petri dish at 4 weeks. (**F**) Ascospores. (**G**) Germ slit (arrowed). *Xylaria vinacea* (SWUF18-2.10). (**H**) Natural habit of stromata. (**I**,**J**) Wrinkled stromata surface with ostioles. (**K**) Ascospores. (**L**) Ascus with ascospores. (**M**) Ascospores with germ slits (arrowed). (**N**) Colony on PDA in a 9 cm Petri dish at 2 weeks. (**O**,**P**) Anamorphs. (**C**,**J**,**K**) by SEM; (**D**,**F**,**G**,**L**,**M**,**O**,**P**) by DIC. Scale bars (**A**,**E**,**H**,**N**) = 1 cm; (**B**,**I**) = 1 mm; (**C**) = 20 µm; (**D**,**F**,**G**,**K**,**M**,**O**,**P**) = 5 µm; (**J**) = 100 µm.

13. *Xylaria thienhirunae* Wangsawat N, Y.-M. Ju, Phosri C, Whalley AJS & Suwannasai N, sp. nov. Figure 9A–G.

MycoBank number: MB 839098.

Etymology—*Thienhirunae* (Lat.): to honour Dr. Surang Thienhirun, a long-term Thai mycologist who contributed to studies of Thai Xylariaceae.

Type–Thailand, Thung Na Lao sub-district, Khon San District, Chaiyaphum province, on termite nests, June 2017, Wangsawat N. SWUF17-44.1 (cultured) (holotype), GenBank accession: ITS = MT622771, α-act = MW459212; Dong Klang sub-district, Khon San District, Chaiyaphum province, on termite nests, June 2017, Wangsawat N. SWUF17-1.3; SWUF17-18.1; SWUF17-18.2; Thung Phra sub-district, Khon San District, Chaiyaphum province, on termite nests, May 2016, Khaeng-raeng R. SWUF16-6.2 (cultured), GenBank accession: ITS = MT622770, α-act = MW459210, β-tub = MW459235; Thung Phra sub-district, Khon San District, Chaiyaphum province, on termite nests, May 2016, Phosri C. SWUF16-7.2 (cultured), GenBank accession: ITS = MT622772; Thung Na Lao sub-district, Khon San District, Chaiyaphum province, on termite nests, May 2016, Phosri C. SWUF16-10.1 (cultured), GenBank accession: ITS = MT622773, α-act = MW459211.

Stromata—Cylindrical, unbranched, with an acuminate apex (0.5–0.9 cm long), 3–9 cm in total length above ground by 1–3 mm broad, 1.2–1.7 cm long at fertile parts, 1.4–6.5 cm long at stipes, with a tortuose rooting base. The surface with conspicuous to inconspicuous perithecial mounds, longitudinally wrinkled, continuous, dark brick (6), black, becoming slightly blackish-brown on the apex, black and smooth on the stipes, texture woody, interior pale luteous (11). Perithecia globose to obovoid, 0.5–0.8 mm diameter by 0.8–1 mm high. Ostioles conic-papillate, black, 0.1 mm broad at base. Asci with eight ascospores, cylindrical, 42–49 µm total length by 4.5–5.6 µm broad, the spore-bearing parts 30–36 µm long, stipes 8.8–16.6 µm long, with apical ring bluing in Melzer’s iodine reagent, inverted hat-shaped, 0.9–1.4 µm high by 0.9–1.5 µm broad.

Ascospores—Light brown to dark brown, unicellular, lemon-shaped, nearly equilateral with narrowly rounded, frequently pinched at the ends, smooth, 4–5.2(–5.7) × 2.7–4 µm, with a straight germ slit spore-length.

Culture characteristics—Colonies reaching the edge of a 9 cm of Petri dish in 4 weeks, at first white and loose-cottony, becoming cream, mostly submerged, with diffuse margin. Reverse remaining uncoloured. Stromata cylindrical, tapering upward, unbranched to branched, flexuous, up to 8 cm long by 1–2 mm diameter, dispersed small stromata, white, becoming cream. Anamorph not observed.

Note—*Xylaria thienhirunae* is similar to *X. chaiyaphumensis* in having similarly-sized lemon-shaped ascospores. However, *X. thienhirunae* differs from *X. chaiyaphumensis* in having larger perithecia (up to 0.8–1 mm high) and half-exposed perithecial mounds (ca. 0.1 mm broad at base). There are 2–3 ostioles/mm in *X. thienhirunae*, while there are 3–4 ostioles/mm in *X. chaiyaphumensis*. In addition, the asci of *X. thienhirunae* have shorter stipes of asci (only 8.8–16.6 µm long), and its ascospores are frequently pinched at the ends. The germ slit of *X. thienhirunae* ascospores is straight and full length, while the germ slit of the *X. chaiyaphumensis* ascospores is half of the spore length.

14. *Xylaria vinacea* Wangsawat N, Y.-M. Ju, Phosri C, Whalley AJS & Suwannasai N, sp. nov. Figure 9H–P.

MycoBank number: MB 839106.

Etymology—*Vinacea* (Lat.): referring to the vinaceous colour of mycelium in culture medium.

Type—Thailand, Nong Lat sub-district, Waritchaphum District, Sakon Nakhon province, on termite nests, July 2018, Wangsawat N. SWUF18-2.10 (cultured) (holotype), GenBank accession: ITS = MT622783, α-act = MW459220, β-tub = MW459240; Phang Khon sub-district, Phanh Khon District, Sakon Nakhon province, on termite nests, July 2018, Phosri C. SWUF18-2.1 (cultured), GenBank accession: ITS = MT622781, α-act = MW459219, β-tub = MW459239; Nong Lat sub-district, Waritchaphum District, Sakon Nakhon province, on termite nests, July 2018, Phosri C. SWUF18-2.3 (cultured), GenBank accession: ITS = MT622782; Thung Phra sub-district, Khon San District, Chaiyaphum province, on termite nests, June 2017, Wangsawat N. SWUF17-7.

Stromata—Fusoid to cylindrical, unbranched, with acuminate apex, 3–5 cm in total length above ground by 2.5–3 mm broad, 1.6–3.5 cm long at fertile parts, 0.9–3.3 cm long at stipes. Surface with conspicuous to mostly slight perithecial mounds, wrinkled, dark brick (60), black and smooth stipes, texture woody, interior white to cream. Perithecia obovoid, 0.3–0.5 mm diameter by 0.3–0.7 mm high. Ostioles papillate, black, 0.2–0.3 mm broad at base. Asci with eight spores, cylindrical, 69.5–120 µm total length by 3.8–5.2 µm broad, the spore-bearing parts 35–59 µm long, stipes 24–66.6 µm long, with apical ring bluing in Melzer’s iodine reagent, inverted hat-shaped, 1.2–1.8 µm high by 1.7–2.2(–2.4) µm broad.

Ascospores—Light brown to dark brown, unicellular, ellipsoid, inequilateral with narrowly rounded ends, smooth, 6.7–8 × 2.8–3.7 µm, with straight, full spore-length to nearly spore-length germ slit on flattened side.

Culture characteristics—Colonies on OA and PDA mostly similar. Colonies not reaching the edge of a 9 cm Petri dish in 4 weeks, at first white and loose-cottony, becoming brown vinaceous (84), zonate, with brown and white concentric zones, mostly aerial mycelium, with lobed margin. Reverse uncoloured. Immature stromata cylindrical, unbranched, sometimes branched, up to 1.5 cm long by 1–2 mm diameter, black or black-purple, white to grey at tip and upper part of mycelium due to conidial production. Anamorph—Conidiophores in upright, dichotomously branched several times from base, smooth, hyaline. Conidiogenous cells terminal, cylindrical, 20–39 × 1.8–3.2(–3.9) µm, smooth, bearing several terminal and lateral denticulate conidial secession scars. Conidia produced holoblastically in sympodial sequence, hyaline, smooth, obovoid, 3.2–3.9(–4.4) × 2.2–3 µm, with a flattened base indicating former point of attachment to conidiogenous cell.

Note—*Xylaria vinacea* is similar to *X. fimbriata* [6,27] in stromatal shape and ascospore size. However, *X. vinacea* differs from *X. fimbriata* in having an acuminate apex, larger perithecia (0.3–0.5 mm diameter × 0.3–0.7 mm high) and papillate ostioles. *Xylaria fimbriata* has a fimbriate stromatal apex, smaller perithecia (2.5–3 mm diameter) and coarsely papillate to conic-papillate ostioles. The ascospore size of *X. vinacea* is closer to that of *X. kedahae* (7.5–9 × 3–3.5 × 4–4.5 µm) [5], but the stromatal shape and size are different. The stromata of *X. kedahae* have three cylindrical fertile branches, and their diameter reaches up to 4 mm. The ascospore ends of *X. kedahae* are usually minutely pinched, while the ascospores of *X. vinacea* have narrowly rounded ends (Table 2).

## 4. Discussion

In the present study, 12 new taxa of *Xylaria* subgenus *Pseudoxylaria* from northeast Thailand are described and compared with closely related species based on morphological and molecular data. The phylogenetic trees were based on a combined ACT-TUB dataset to support the classification at a supraspecific level [2,28], and complemented by the analysis of an ITS dataset, which is the most commonly used locus for barcoding fungi at the species level [16,29]. Therefore, with the 12 taxa described here for the first time, 17 species and one variety of subgenus *Pseudoxylaria* are now known in Thailand, i.e., *X. acuminatilongissima*, *X. atrodivaricata*, *X. chaiyaphumensis*, *X. conica*, *X. escharoidea*, *X.* cf. *escharoidea*, *X. fulvescens*, *X. ischnostroma*, *X. margaretae*, *X. minima*, *X. nigripes*, *X*. cf. *nigripes*, *X. reinkingii* var. *microspora*, *X. siamensis*, *X. sihanonthii*, *X. subintraflava*, *X. tanganyikaensis*, *X. thienhirunae*, and *X. vinacea* [9,10].

*Pseudoxylaria* was originally proposed by Boedijn [30] as a genus to separate *Xylaria nigripes* from all other species of *Xylaria*. Since all *Xylaria* species from termite nests form a distinct clade within *Xylaria* [2,31,32,33], a case could be made to transfer them to the genus *Pseudoxylaria*. Nevertheless, we prefer to consider *Pseudoxylaria* a subgenus of *Xylaria* due to the fact that there are no definite morphological characteristics to warrant the separation between species of *Pseudoxylaria* and *Xylaria*. Previously, all species of *Pseudoxylaria* seemed to share a tiny ascospore size shorter than 8 μm, but with the discovery of *X. conica* and *X. ischnostroma* in the present study, which have ascospores longer than 10 μm, this trait seems no longer useful to separate *Pseudoxylaria* and *Xylaria*. In addition, some species of *Pseudoxylaria*, such as *X. guepini*, *X. coprinicola*, and *X. ripicola*, are not associated with termite nests but soil. Last but not least, we believe that we only know a small fraction of *Xylaria* species associated with termite nests, and therefore additional studies are necessary to confirm that all species are nested inside *Pseudoxylaria*.

Thailand is located within the tropical climatic zone with various types of forest ecosystems that are suitable for termite growth. Termite nests can be easily found in forests, farmlands, and rural shelters. The whole family Macrotermitinae includes up to 12 genera and 373 species worldwide [34], but in Thailand, five genera (*Ancistrotermes*, *Hypotermes*, *Macrotermes*, *Microtermes* and *Odontotermes*) and 46 species of termites in the subfamily Macrotermitinae can be found throughout the country [35]. Despite most of the diversity of the termite subfamily Macrotermitinae being located in Africa, only three species of *Xylaria* subgenus *Pseudoxylaria* have been documented from that continent: *X. arenicola*, *X. furcata* var. *hirsuta*, and *X. escharoidea*. In contrast, approximately 40 species of *Xylaria* subgenus *Pseudoxylaria* are known in Asia, where much less diversity of Macrotermitinae has been reported, suggesting that African species of *Xylaria* associated with termite nests are severely understudied. Results of the present study have shown that the stromata of *Xylaria* species are diverse in shape and size as well as in their perithecial surface and cultural characteristics. The ascospores size ranges from 4 to 19 µm long, and they vary in colour from light brown to dark brown. These results agree with those reported by Hsieh et al. [2]. The morphology of the teleomorph and anamorph states, as well as the cultural characteristics, of *Xylaria* species associated with termite nests, are apparently highly diverse, to such a degree that is nearly comparable with the morphological diversification found among *Xylaria* species associated with other substrates [2], in accordance with the phylogenetic structure inferred from DNA data.

In the present study, we found two species, *X*. cf. *escharoidea* and *X*. cf. *nigripes*, closely resembling *X. escharoidea* and *X. nigripes*, respectively, which are widely distributed throughout Asia and elsewhere [5]. The morphological characteristics of our species are similar to the descriptions of *X. escharoidea* and *X. nigripes*, but the nucleotide sequences showed differences, producing longer branches in the phylogenetic tree. Although both *X. escharoidea* and *X. nigripes* were previously recorded in Thailand [9,10], no cultures were obtained. To clarify these problems, more samples from different locations are required for further investigation.

## 5. Conclusions

The currently known diversity of *Xylaria* subgenus *Pseudoxylaria* in Thailand consists in 17 species and one variety, representing 43% of the 40 species known in the world. Nonetheless, this high diversity likely represents only a small fraction of the subgenus *Pseudoxylaria* in Thailand because the investigations were conducted mainly in the northeast region. By now, all species of *Xylaria* associated with termite nests belong to *Pseudoxylaria*, but the only known synapomorphic trait of this clade, the small ascospore size <8 µm, is not present in all species, suggesting that additional studies are necessary to confirm its most suitable taxonomic status.

## Figures and Tables

**Table 1:** Taxa used in this study.

Species	Collection Number	Country	GenBank Accession Number ITS	Reference	GenBank Accession Number Beta-Tubulin	Reference	GenBank Accession Number Alpha-Actin	Reference
*Astrocystis bambusae*	HAST 89021904	Taiwan	-	-	GQ495942	[2]	GQ449239	[2]
*A. mirabilis*	HAST 94070803	Taiwan	-	-	GQ495941	[2]	GQ449238	[2]
*A. sublimbata*	HAST 89032207	Taiwan	-	-	GQ495940	[2]	GQ449236	[2]
*Kretzschmaria* *clavus*	JDR 114	French Guiana	-	-	EF025611	[2]	EF025596	[2]
*K. guyanensis*	HAST 89062903	Taiwan	GU300079	[2]	GQ478214	[2]	GQ408901	[2]
*K. sandvicensis*	JDR 113	USA, Hawaiian Islands	-	-	GQ478211	[2]	GQ398234	[2]
*Nemania bipapillata*	HAST 90080610	Taiwan	-	-	GQ470221	[2]	GQ389693	[2]
*N. diffusa*	HAST 91020401	Taiwan	-	-	GQ470220	[2]	GQ389692	[2]
*N. illita*	JDR 236	USA	-	-	EF025608	[2]	EF025593	[2]
*Poronia pileiformis*	WSP 88113001	Taiwan	GU324760	[2]	GQ502720	[2]	GQ455449	[2]
*Rosellinia buxi*	JDR 99	France	-	-	GQ470228	[2]	GQ398228	[2]
*R. necatrix*	HAST 89062904	Taiwan	-	-	EF025603	[2]	EF025588	[2]
*Xylaria acuminatilongissima*	HAST 623	Taiwan	EU178738	[6]	GQ502711	[2]	GQ853046	[2]
*X. adscendens*	JDR 865	Thailand	GU322432	[2]	GQ487709	[2]	GQ438746	[2]
*X. apoda*	HAST 90080804	Taiwan	GU322437	[2]	GQ495930	[2]	GQ438751	[2]
*X. arbuscula* var. *plenofissura*	HAST 93082814	Taiwan	GU339495	[2]	GQ478225	[2]	GQ421285	[2]
*X. atrodivaricata*	HAST 95052001	Taiwan	EU178739	[6]	GQ502713	[2]	GQ853048	[2]
*X. atrosphaerica*	HAST 91111214	Taiwan	GU322459	[2]	GQ495953	[2]	GQ452363	[2]
*X. bambusicola*	JDR 162	Thailand	GU300088	[2]	GQ478223	[2]	GQ408910	[2]
*X. brunneovinosa*	HAST 720	Taiwan	EU179862	[6]	GQ502706	[2]	GQ853041	[2]
*X. chaiyaphumensis*	SWUF16-04.1	Thailand	MT622777	This study	-	-	-	-
*X. chaiyaphumensis*	SWUF16-11.4	Thailand	MT622776	This study	MW459236	This study	MW459213	This study
*X. chaiyaphumensis*	SWUF17-15.1	Thailand	MT622774	This study	-	-	MW459214	This study
*X. chaiyaphumensis*	SWUF17-49.2	Thailand	MT622775	This study	-	-	MW459215	This study
*X. cirrata*	HAST 664	Taiwan	EU179863	[6]	GQ502707	[2]	GQ853042	[2]
*X. coccophora*	HAST 786	French Guiana	GU300093	[2]	GQ487701	[2]	GQ421289	[2]
*X. conica*	SWUF18-4.3	Thailand	MT622786	This study	-	-	MW459223	This study
*X. conica*	SWUF18-4.4	Thailand	MT622787	This study	MW459243	This study	MW459224	This study
*X. coprinicola*	1145	China	HM585020	[23]	HM585018	[23]	HM585017	[23]
*X. crozonensis*	HAST 398	France	GU324748	[2]	GQ502697	[2]	GQ455441	[2]
*X. cubensis*	GENT 159	Papua New Guinea	-	-	GQ502702	[2]	GQ455446	[2]
*X. cubensis*	HAST 515	French West Indies	GU373810	[2]	GQ502701	[2]	GQ455445	[2]
*X. culleniae*	JDR 189	Thailand	GU322442	[2]	GQ495935	[2]	GQ438756	[2]
*X. curta*	HAST 92092022	Taiwan	GU322443	[2]	GQ495936	[2]	GQ438757	[2]
*X. escharoidea*	HAST 658	Taiwan	EU179864	[6]	GQ502709	[2]	GQ853044	[2]
*X. escharoidea*	CLZhao TJ-1	China	MK343687	Unpublished	-	-	-	-
*X. escharoidea*	CLZhao TJ-2	China	MK343688	Unpublished	-	-	-	-
*X. escharoidea*	CLZhao TJ-3	China	MK343689	Unpublished	-	-	-	-
*X. escharoidea*	SWFU000021	China	MK862248	Unpublished	-	-	-	-
*X. escharoidea*	274-CW107	China	KU194333	Unpublished	-	-	-	-
*X. escharoidea*	TF18	China	MN509048	Unpublished	-	-	-	-
*X. escharoidea*	Y-4	China	KC462194	Unpublished	-	-	-	-
*X.* cf. *escharoidea*	SWUF17-35.1	Thailand	MT622792	This study	MW459227	This study	MW459200	This study
*X.* cf. *escharoidea*	SWUF17-38.1	Thailand	MT622793	This study	-	-	-	-
*X.* cf. *escharoidea*	SWUF17-39.1	Thailand	MT622794	This study	MW459228	This study	MW459201	This study
*X.* cf. *escharoidea*	SWUF17-39.2	Thailand	MT622795	This study	-	-	-	-
*X. fimbriata*	HAST 491	French West Indies	GU324753	[2]	GQ502705	[2]	GQ853040	[2]
*X. fulvescens*	SWUF17-27.2	Thailand	MT622780	This study	MW459238	This study	MW459218	This study
*X. griseosepiacea*	HAST 641	Taiwan	EU179865	[6]	GQ502714	[2]	GQ853049	[2]
*X. hypoxylon*	HAST 95082001	Taiwan	GU300095	[2]	GQ487703	[2]	GQ427195	[2]
*X. ianthinovelutina*	HAST 553	French West Indies	GU322441	[2]	GQ495934	[2]	GQ438755	[2]
*X. insolita*	HAST 99090301-1251	Taiwan	MN655979	[8]	MN656983	[8]	MN656985	[8]
*X. intracolorata*	HAST 90080402	Taiwan	GU324741	[2]	GQ502690	[2]	GQ452375	[2]
*X. intraflava*	HAST 725	Taiwan	EU179866	[6]	GQ502718	[2]	GQ853053	[2]
*X. ischnostroma*	SWUF18-22.1	Thailand	MT622788	This study	MW459244	This study	MW459225	This study
*X. juruensis*	HAST 92042501	Taiwan	GU322439	[2]	GQ495932	[2]	GQ438753	[2]
*X. laevis*	HAST 95,072,910	Taiwan	GU324747	[2]	GQ502696	[2]	GQ455440	[2]
*X. luteostromata* var. *macrospora*	HAST 508	French West Indies	GU324739	[2]	GQ502688	[2]	GQ452373	[2]
*X. margaretae*	SWUF17-34.1	Thailand	MT622778	This study	-	-	MW459216	This study
*X. margaretae*	SWUF17-34.2	Thailand	MT622779	This study	MW459237	This study	MW459217	This study
*X. minima*	SWUF18-3.2	Thailand	MT622789	This study	MW459245	This study	MW459226	This study
*X. multiplex*	HAST 580	French West Indies	GU300098	-	GQ487705	[2]	GQ427198	[2]
*X. nigripes*	HAST 653	Taiwan	GU324755	[2]	GQ502710	[2]	GQ853045	[2]
*X. nigripes*	Xn1	China	MK748600	Unpublished	-	-	-	-
*X. nigripes*	Xn-6	China	JQ967448	Unpublished	-	-	-	-
*X. nigripes*	Xn-7	China	JQ979095	Unpublished	-	-	-	-
*X. nigripes*	CWF	Taiwan	KJ627787	Unpublished	-	-	-	-
*X. nigripes*	420526MF0342	China	MG712340	Unpublished	-	-	-	-
*X. nigripes*	Hf-1A	Taiwan	JQ927570	Unpublished	-	-	-	-
*X. nigripes*	4-29	China	HM050414	Unpublished	-	-	-	-
*X. nigripes*	ZD17052101	China	MN523323	Unpublished	-	-	-	-
*X.* cf. *nigripes*	SWUF17-31.2	Thailand	MT622790	This study	MW459229	This study	MW459202	This study
*X.* cf. *nigripes*	SWUF17-36.1	Thailand	MT622791	This study	-	-	MW459203	This study
*X. ochraceostroma*	HAST 401	Taiwan	EU179869	[6]	GQ502717	[2]	GQ853052	[2]
*X. oligotoma*	HAST 784	French Guiana	GU300092	[2]	GQ487700	[2]	GQ421288	[2]
*X. ophiopoda*	HAST 93082805	Taiwan	GU322461	[2]	GQ452365	[2]	GQ495955	[2]
*X. plebeja*	HAST 91122401	Taiwan	GU324740	[2]	GQ502689	[2]	GQ452374	[2]
*X. polymorpha*	JDR1012	USA	GU322460	[2]	GQ495954	[2]	GQ452364	[2]
*X. reinkingii* var. *microspora*	SWUF17-19.1	Thailand	MT622769	This study	MW459234	This study	MW459209	This study
*X. ripicola*	KA11-0060-1	South Korea	NR153251	[24]	-	-	-	-
*X. ripicola*	KA11-0060-2	South Korea	KM817200	[24]	-	-	-	-
*X. siamensis*	SWUF17-20.2	Thailand	MT622765	This study	MW459233	This study	MW459208	This study
*X. siamensis*	SWUF17-20.3	Thailand	MT622766	This study	-	-	-	-
*X. siamensis*	SWUF17-20.4	Thailand	MT622767	This study	-	-	-	-
*X. siamensis*	SWUF17-20.6	Thailand	MT622768	This study	-	-	-	-
*X. sihanonthii*	SWUF18-5.1	Thailand	MT622784	This study	MW459241	This study	MW459221	This study
*X. sihanonthii*	SWUF18-1.3	Thailand	MT622785	This study	MW459242	This study	MW459222	This study
*X. striata*	HAST 304	Taiwan	GU300089	[2]	GQ478224	[2]	GQ421284	[2]
*X. subescharoidea*	HAST 660	Taiwan	GU324754	[2]	GQ502708	[8]	GQ853043	[8]
*X. subescharoidea*	HAST 99060401-1188	Taiwan	MN655980	[8]	MN656984	[8]	MN656986	[8]
*X. subintraflava*	SWUF16-4.3	Thailand	MT622762	This study	MW459230	This study	MW459204	This study
*X. subintraflava*	SWUF16-11.1	Thailand	MT622763	This study	-	-	MW459205	This study
*X. subintraflava*	SWUF17-9.2	Thailand	MT622758	This study	-	-	-	-
*X. subintraflava*	SWUF17-13.1	Thailand	MT622759	This study	-	-	-	-
*X. subintraflava*	SWUF17-22.2	Thailand	MT622764	This study	MW459231	This study	MW459206	This study
*X. subintraflava*	SWUF17-24.2	Thailand	MT622757	This study	MW459232	This study	MW459207	This study
*X. subintraflava*	SWUF18-9.1	Thailand	MT622760	This study	-	-	-	-
*X. subintraflava*	SWUF18-9.2	Thailand	MT622761	This study	-	-	-	-
*X. terricola*	YMJ 1375	Taiwan	MF577042	[25]	MF577044	[25]	MF577045	[25]
*X. thienhirunae*	SWUF16-6.2	Thailand	MT622770	This study	MW459235	This study	MW459210	This study
*X. thienhirunae*	SWUF16-7.2	Thailand	MT622772	This study	-	-	-	-
*X. thienhirunae*	SWUF16-10.1	Thailand	MT622773	This study	-	-	MW459211	This study
*X. thienhirunae*	SWUF17-44.1	Thailand	MT622771	This study	-	-	MW459212	This study
*X. venustula*	HAST 88113002	Taiwan	GU300091	[2]	GQ487699	[2]	GQ421287	[2]
*X. vinacea*	SWUF18-2.1	Thailand	MT622781	This study	MW459239	This study	MW459219	This study
*X. vinacea*	SWUF18-2.3	Thailand	MT622782	This study	-	-	-	-
*X. vinacea*	SWUF18-2.10	Thailand	MT622783	This study	MW459240	This study	MW459220	This study
*X.* sp. 1	HAST 642	Taiwan	GU324759	[2]	GQ502719	[2]	GQ853054	[2]
*X.* sp. 3	HAST 722	Taiwan	GU324756	[2]	GQ502712	[2]	GQ853047	[2]
*X.* sp. 4	HAST 646	Taiwan	GU324757	[2]	GQ502715	[2]	GQ853050	[2]
*X.* sp. 5	HAST 650	Taiwan	GU324758	[2]	GQ502716	[2]	GQ853051	[2]
*Xylaria* sp.	ASMC3	Vietnam	EU164404	[22]	-	-	-	-
*Xylaria* sp.	AAG5	Africa	EU164400	[22]	-	-	-	-
*Xylaria* sp.	HNHB	China	FN812862	Unpublished	-	-	-	-
*Xylaria* sp.	D12	China	KC414236	Unpublished	-	-	-	-
*Xylaria* sp.	TF37	China	MN526593	Unpublished	-	-	-	-
*Xylaria* sp.	4-E7-2	China	FN812842	Unpublished	-	-	-	-
*Xylariaceae* sp.	342.D	South Africa	FJ425676	[26]	-	-	-	-
*Xylariaceae* sp.	341	South Africa	FJ425675	[26]	-	-	-	-

**Table 2:** A key to species of *Xylaria* associated with termite nests and soil in Thailand.

1. Ascospores with a median germ pore, (3.6–)3.9–4.7(–5) × (2.35–)2.5–3.2(–3.5) µm	*X. escharoidea*
1. Ascospores with a germ slit	2
2. Stromata usually repeatedly branched, with prominent perithecial mounds, ascospores shorter than 6 µm	3
2. Stromata unbranched or sparingly branched, perithecia naked or presenting either inconspicuous or conspicuous perithecial mounds	4
3. Stromatal surface white when immature, becoming blackish at maturity; ascospores ellipsoid-inequilateral, germ slit straight spore-length or nearly on flattened side, 5.0–6.0 × 2.5–3.5 µm	*X. siamensis*
3. Stromatal surface dull coloured, becoming blackish at maturity; ascospores short fusoid-inequilateral, 3.5–5.0 × 2.0–3.0 µm	*X. atrodivaricata* *
4. Ascospores > 10 µm, germ slit straight full spore-length	5
4. Ascospores < 10 µm, germ slit straight 3/4 or full spore-length	7
5. Stromal surface whitish to greyish with black ostioles; ascospores blackish-brown,(11–)14.0–19.4 × (6.5–)7.0–10.0 µm	*X. tanganyikaensis* *
5. Stromatal surface blackish-brown to black with black ostioles; ascospores light brown, brown to dark brown, frequently < 14 µm	6
6. Perithecia immersed, usually with prominent perithecial mounds; ascospores ellipsoid-inequilateral with narrowly rounded ends, (10–)10.8–12.3(–13.2) × 4.5–6(–6.4) µm; apical apparatus 4–4.5 × 2–3 µm	*X. conica*
6. Perithecia naked or nearly so; ascospores ellipsoid-inequilateral with narrowly rounded ends, some pinched at the ends,10.3–11.6(–12) × 4.8–5.6 µm; apical apparatus 1.5–2 × 2–3 µm	*X. ischnostroma*
7. Stromatal surface white at maturity; ascospores 5.4–6(–6.3) × 2.4–3.0 µm, straight germ slit nearly spore-length on convex side	*X. reinkingii* var. *microspora*
7. Stromatal surface other than white, usually dull coloured at maturity	8
8. Stromata usually more or less cylindrical, often exceeding 3 mm in diameter	9
8. Stromata usually slender, fusiform to cylindrical, rarely exceeding 3 mm in diameter	12
9. Stromatal surface blackish-brown to black or dark brick; ascospores mostly longer than 5 µm	10
9. Stromatal surface ochraceous to fawn, luteous, greyish or dull black; ascospores mostly shorter than 5 µm	11
10. Perithecia presenting very conspicuous mounds, blackish-brown; ostioles conic-papillate; ascospores 7.5–9.5 × (3.2–)3.5–4.5 µm	*X. sihanonthii*
10. Perithecia immersed, brown; ostioles papillate; ascospores 6.7–8 × 2.8–3.7 µm	*X. vinacea*
11. Stromata acuminate at the apex, unbranched, ochraceous to yellowish-brown on surface; ascospores inequilateral, 4–5 × 1.8–2.5 µm	*X. acuminatilongissima* *
11. Stromata usually blunt or, infrequently, mucronate at the apex, greyish-brown on surface; ascospores slightly inequilateral to nearly equilateral, 3.5–5 × 2–3 µm	X. nigripes *
12. Ascospores mostly > 5 µm	13
12. Ascospores mostly < 5 µm	14
13. Stromata very thin, 0.5–1 mm broad; perithecia naked or so on, hairy; ascospores 5.8–6.8 × 2.7–3.2 µm	*X. minima*
13. Stromata slender, perithecia immersed, forming conspicuous mounds, without hair; ascospores (4.5–)5–6.2 × 2.2–3 µm	*X. fulvescens*
14. Ascospores short, fusoid, pinched at the ends	15
14. Ascospores ellipsoid-inequilateral to nearly equilateral with narrowly rounded ends	16
15. Perithecia 0.3–0.7 mm diameter, 3–4 ostioles/mm; ostioles conic-papillate; ascospores fusoid with pinched ends, 4–5(–5.7) × (2.5–)3–3.8 µm; germ slit of half-full ascospore length	*X. chaiyaphumensis*
15. Perithecia 0.5–0.8 mm diameter, 2–3 ostioles/mm; ostioles conic-papillate; ascospores fusoid with pinched ends, 4–5.2(–5.7) × 2.7–4 µm; germ slit full ascospore length	*X. thienhirunae*
16. Stromatal surface longitudinally wrinkled with long stipes, unbranched with fertile parts; ascospores 3.5–5 × 1.8–2.5 µm	*X. subintraflava*
16. Stromatal surface wrinkled with acuminate at apex, without fertile parts, unbranched or two-branched at apex; ascospores (3.2–)3.8–5 × (1.8–)2–2.5 µm	*X. margaretae*

* See Srihanant and Petcharat [10].

## Data Availability

Publicly available datasets were analysed in this study. This data can be found here: https://www.ncbi.nlm.nih.gov/, accessed on 16 June 2020; https://www.mycobank.org/page/Type%20specimens%20search, accessed on 23 March 2021; https://www.github.com/niwanawangsawat/Aligment-sequence-files.git, accessed on 17 June 2021.

## Supplementary Materials

The following are available online at https://www.mdpi.com/article/10.3390/biology10070575/s1, Figure S1: Phylogenetic tree inferred from ML analysis based on a dataset of ACT-TUB sequences of *Xylaria* species associated with termite nests. Branches are labelled with likelihood bootstrap support 1000 replications. *Poronia pileiformis* (WSP 88113001) is the outgroup. Figure S2: Phylogenetic tree inferred from BI analysis based on a dataset of exon ACT-TUB sequences of *Xylaria* species and related genera. Branches are labelled with Bayesian posterior probabilities and bootstrap values from BI and ML analyses, respectively. *Poronia pileiformis* (WSP 88113001) is the outgroup. Figure S3: Phylogenetic tree inferred from BI analysis based on a dataset of intron ACT-TUB sequences of *Xylaria* species and related genera. Branches are labelled with Bayesian posterior probabilities and bootstrap values from BI and ML analyses, respectively. *Poronia pileiformis* (WSP 88113001) is the outgroup. Figure S4: Phylogenetic tree inferred from ML analysis based on a dataset of ITS sequences of *Xylaria* species associated with termite nests. Branches are labelled with likelihood bootstrap support 1000 replications. *Poronia pileiformis* (WSP 88113001) is the outgroup.

## References

[1] Batra L.R., Batra S.W.T., Batra L.R., Montclair N.J. (1979). Termite-fungus mutualism. Insect-Fungus Symbiosis-Nutrition, Mutualism and Commensalism.

[2] Hsieh H.-M., Lin C.-R., Fang M.-J., Rogers J.D., Fournier J., Lechat C., Ju Y.-M. (2010). Phylogenetic status of *Xylaria* subgenus *Pseudoxylaria* among taxa of the subfamily Xylarioideae (*Xylariaceae*) and phylogeny of the taxa involved in the subfamily. Mol. Phylogenet. Evol..

[3] Petch T. (1913). Termite fungi: A resume. Ann. Roy. Bot. Gard..

[4] Dennis R.W.G. (1961). Xylarioideae and Thamnomycetoideae of Congo. Bull. Jard. Bot. l’État Brux..

[5] Rogers J.D., Ju Y.-M., Lehmann J. (2005). Some *Xylaria* species on termite nests. Mycologia.

[6] Ju Y.-M., Hsieh H.-M. (2007). *Xylaria* species associated with nests of *Odontotermes formosanusin* Taiwan. Mycologia.

[7] Rogers J.D. (1979). The *Xylariaceae*: Systematic, Biological and Evolutionary Aspects. Mycologia.

[8] Hsieh H.-M., Chou J.-C., Ju Y.-M. (2020). *Xylaria* insolita and *X. subescharoidea*: Two newly described species collected from a termite nesting site in Hua-lien, Taiwan. Bot. Stud..

[9] Thienhirun S. (1997). A preliminary account of the *Xylariaceae* of Thailand. Ph.D. Thesis.

[10] Srihanant N., Petcharat V. (2015). Some *Xylaria* species in oil palm and Pará rubber plantation in southern Thailand. Khon. Kaen. Agri. J..

[11] McKnight K.H., Rayner R.W. (1972). A Mycological Colour Chart. Mycologia.

[12] White T.J., Bruns T., Lee S., Taylor J., Innis M.A., Gelfand D.H., Shinsky J.J., White T.J. (1990). Amplification and direct sequencing of fungal ribosomal RNA genes for phylogenetics. PCR Protocols: A Guide to Methods and Applications.

[13] Glass N.L., Donaldson G.C. (1995). Development of primer sets designed for use with the PCR to amplify conserved genes from filamentous ascomycetes. Appl. Environ. Microbiol..

[14] O’Donnell K., Cigelnik E. (1997). Two divergent intragenomic rDNA ITS2 types within a monophyletic lineage of the fungus *Fusarium* are nonorthologous. Mol. Phylogenet. Evol..

[15] Carbone I., Kohn L.M. (1999). A method for designing primer sets for speciation studies in filamentous ascomycetes. Mycologia.

[16] Suwannasai N., Martín M.P., Phosri C., Sihanonth P., Whalley A.J.S., Spouge J.L. (2013). Fungi in Thailand: A Case Study of the Efficacy of an ITS Barcode for Automatically Identifying Species within the *Annulohypoxylon* and *Hypoxylon* Genera. PLoS ONE.

[17] Edgar R.C. (2004). MUSCLE: Multiple sequence alignment with high accuracy and high throughput. Nucleic Acids Res..

[18] Kumar S., Stecher G., Li M., Knyaz C., Tamura K. (2018). MEGA X: Molecular evolutionary genetics analysis across computing platforms. Mol. Biol. Evol..

[19] Stecher G., Tamura K., Kumar S. (2020). Molecular Evolutionary Genetics Analysis (MEGA) for macOS. Mol. Biol. Evol..

[20] Ronquist F., Teslenko M., Van Der Mark P., Ayres D.L., Darling A., Hoehna S., Larget B., Liu L., Suchard M.A., Huelsenbeck J.P. (2012). MrBayes 3.2: Efficient bayesian phylogenetic inference and model choice across a large model space. Syst. Biol..

[21] Rambaut A. (2014). FigTree v1.4.2, a Graphical Viewer of Phylogenetic Trees. http://tree.bio.ed.ac.uk/software/figtree/0.

[22] Guedegbe H.J., Miambi E., Pando A., Houngnandan P., Rouland-Lefevre C. (2009). Molecular diversity and host specificity of termite-associated *Xylaria*. Mycologia.

[23] Ju Y.-M., Hsieh H.-M., He X.-S. (2011). *Xylaria coprinicola*, a new species that antagonizes cultivation of *Coprinus comatus* in China. Mycologia.

[24] Kim C.S., Jo J.W., Kwag Y.-N., Oh S.-O., Lee S.-G., Sung G.-H., Han J.-G., Oh J., Shrestha B., Kim S.-Y. (2016). New Records of *Xylaria* Species in Korea: *X. ripicola* sp. nov. and *X. tentaculata*. Mycobiology.

[25] Chou W.-N., Hsieh H.-M., Ju Y.-M. (2017). *Xylaria terricola* sp. nov., a terrestrial anamorphic *Xylaria* species found in Taiwan. Fungal Sci..

[26] Visser A.A., Ros V.I., De Beer Z.W., Debets A.J., Hartog E., Kuyper T.W., Læssoe T., Slippers B., Aanen D.K. (2009). Levels of specificity of *Xylaria* species associated with fungus-growing termites: A phylogenetic approach. Mol. Ecol..

[27] Lloyd C.G. (1917). Mycological notes 51. Mycol. Writ..

[28] Hsieh H.-M., Ju Y.-M., Rogers J.D. (2005). Molecular phylogeny of *Hypoxylon* and closely related genera. Mycologia.

[29] Liu Z.-B., Zhou M., Yuan Y., Dai Y.-C. (2021). Global Diversity and Taxonomy of *Sidera* (*Hymenochaetales*, *Basidiomycota*): Four New Species and Keys to Species of the Genus. J. Fungi.

[30] Boedijn K.B. (1959). On a new family of the Sphaeriales. Persoonia.

[31] U’Ren J.M., Miadlikowska J., Zimmerman N.B., Lutzoni F., Stajich J.E., Arnold A.E. (2016). Contributions of North American endophytes to the phylogeny, ecology, and taxonomy of *Xylaria*ceae (*Sordariomycetes*, *Ascomycota*). Mol. Phylogenet. Evol..

[32] Wendt L., Sir E.B., Kuhnert E., Heitkämper S., Lambert C., Hladki A.I., Romero A.I., Luangsa-Ard J.J., Srikitikulchai P., Persoh D. (2018). Resurrection and emendation of the Hypoxylaceae, recognised from a multigene phylogeny of the *Xylaria*les. Mycol. Prog..

[33] Konta S., Hyde K.D., Phookamsak R., Xu J.C., Maharachchikumbura S.S.N., Daranagama D.A., McKenzie E.H.C., Boonmee S., Tibpromma S., Eungwanichayapant P.D. (2020). Polyphyletic genera in *Xylariaceae* (*Xylariales*): *Neoxylaria* gen. nov. and *Stilbohypoxylon*. Mycosphere.

[34] Krishna K., Grimaldi D.A., Krishna V., Engel M.S. (2013). Treatise on the Isoptera of the world 4. Termitidae (Part one). Bull. Am. Mus. Nat. Hist..

[35] Sornnuwat Y., Vongkaluang C., Takematsu Y. (2004). A systematic key to termites of Thailand. Kasetsart J..

