# Supplementary material for: Twelve New Taxa of Xylaria Associated with Termite Nests and Soil from Northeast Thailand
Source: Biology (Basel). 2021 Jun 23;10(7):575. doi: 10.3390/biology10070575 (PMC8301132; doi:10.3390/biology10070575)
Supplement: Supplementary file 1 [file biology-10-00575-s001.zip › biology-1198903-supplementary.pdf]

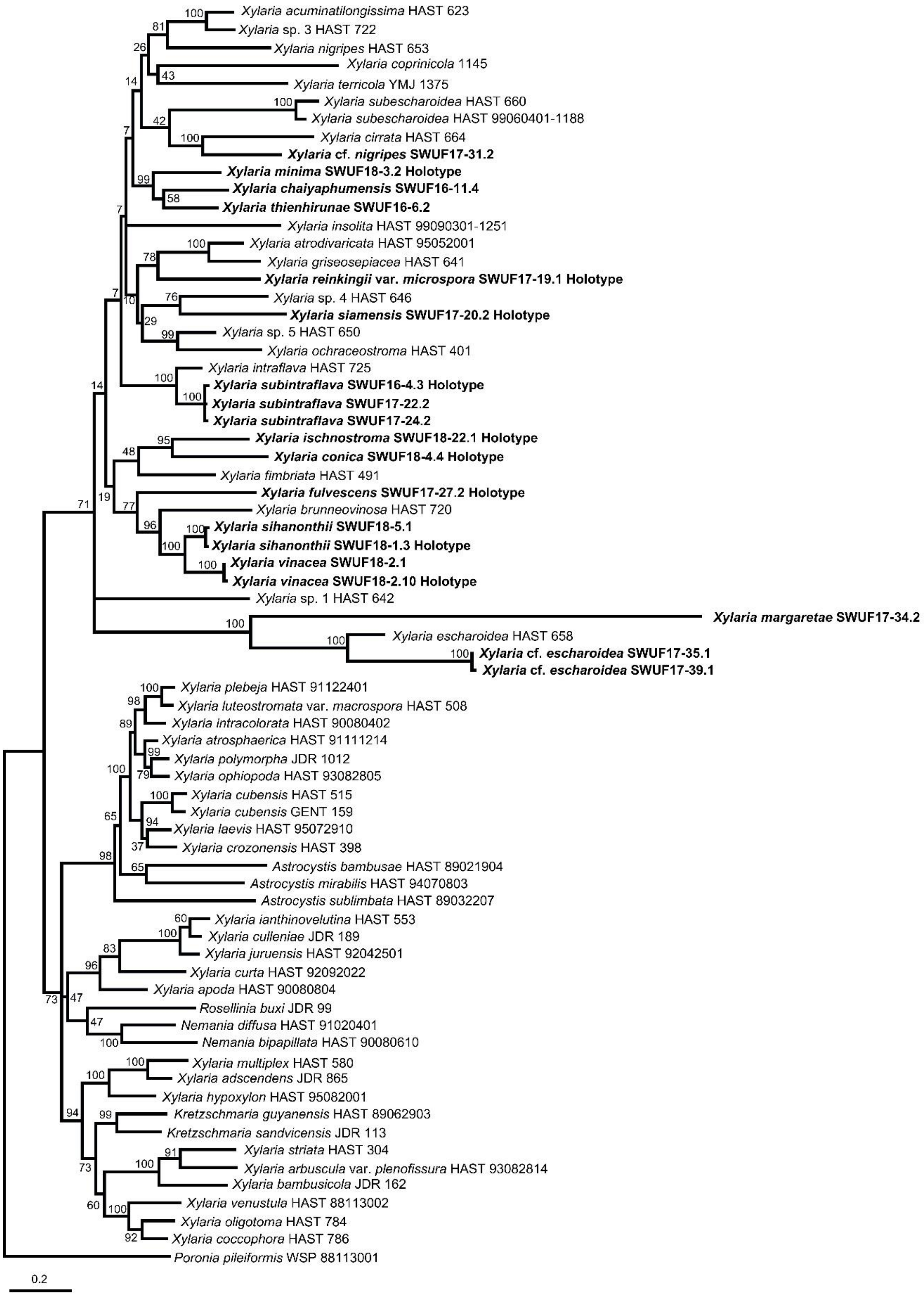

Figure S1. Phylogenetic tree inferred from ML analysis based on a dataset of ACT-TUB sequences of *Xylaria* species associated with termite nests. Branches are labelled with likelihood bootstrap support 1000 replications. *Poronia pileiformis* (WSP 88113001) is the outgroup.

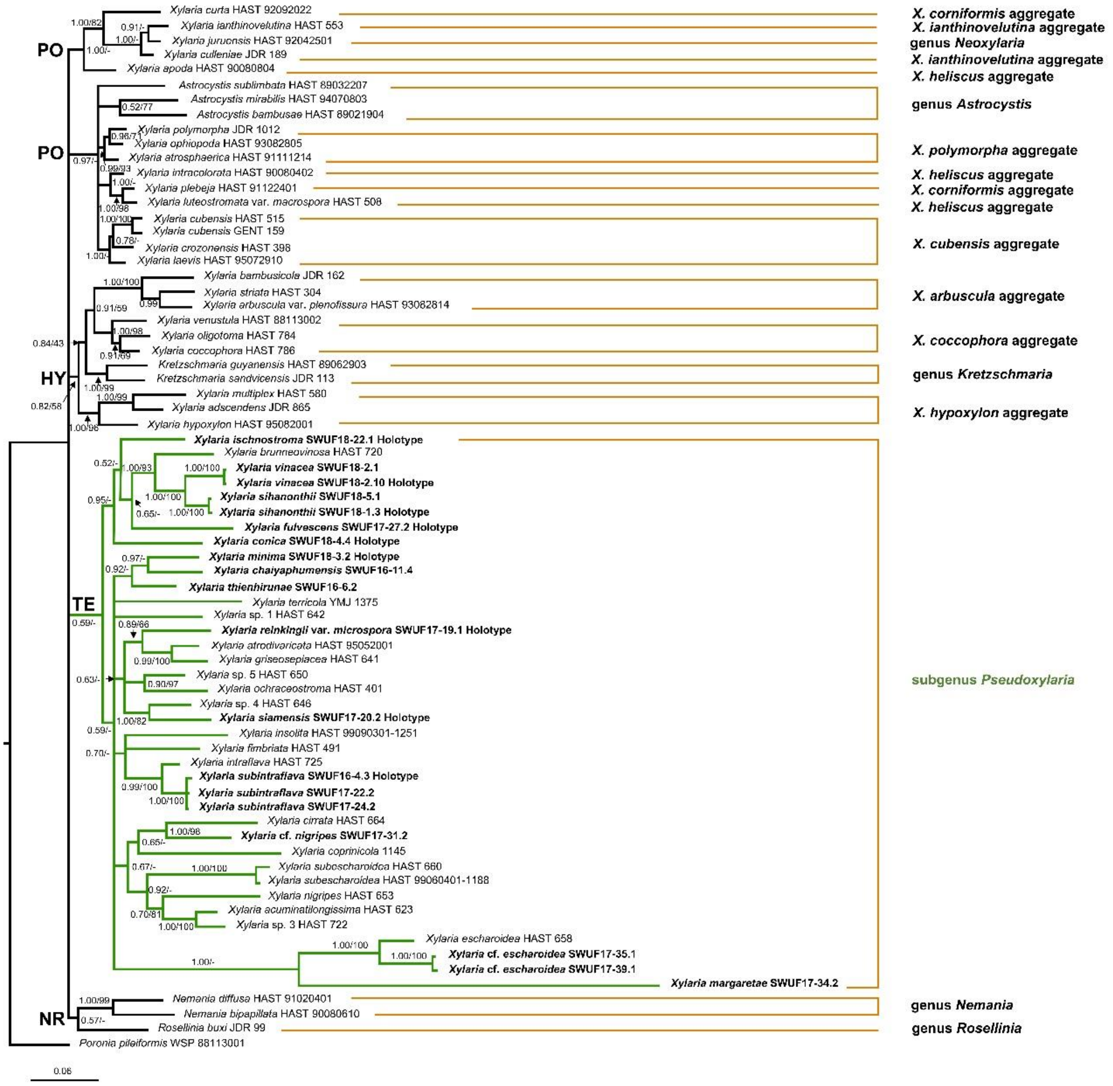

Figure S2. Phylogenetic tree inferred from BI analysis based on a dataset of exon ACT-TUB sequences of *Xylaria* species and related genera. Branches are labelled with Bayesian posterior probabilities and bootstrap values from BI and ML analyses, respectively. *Poronia pileiformis* (WSP 88113001) is the outgroup.

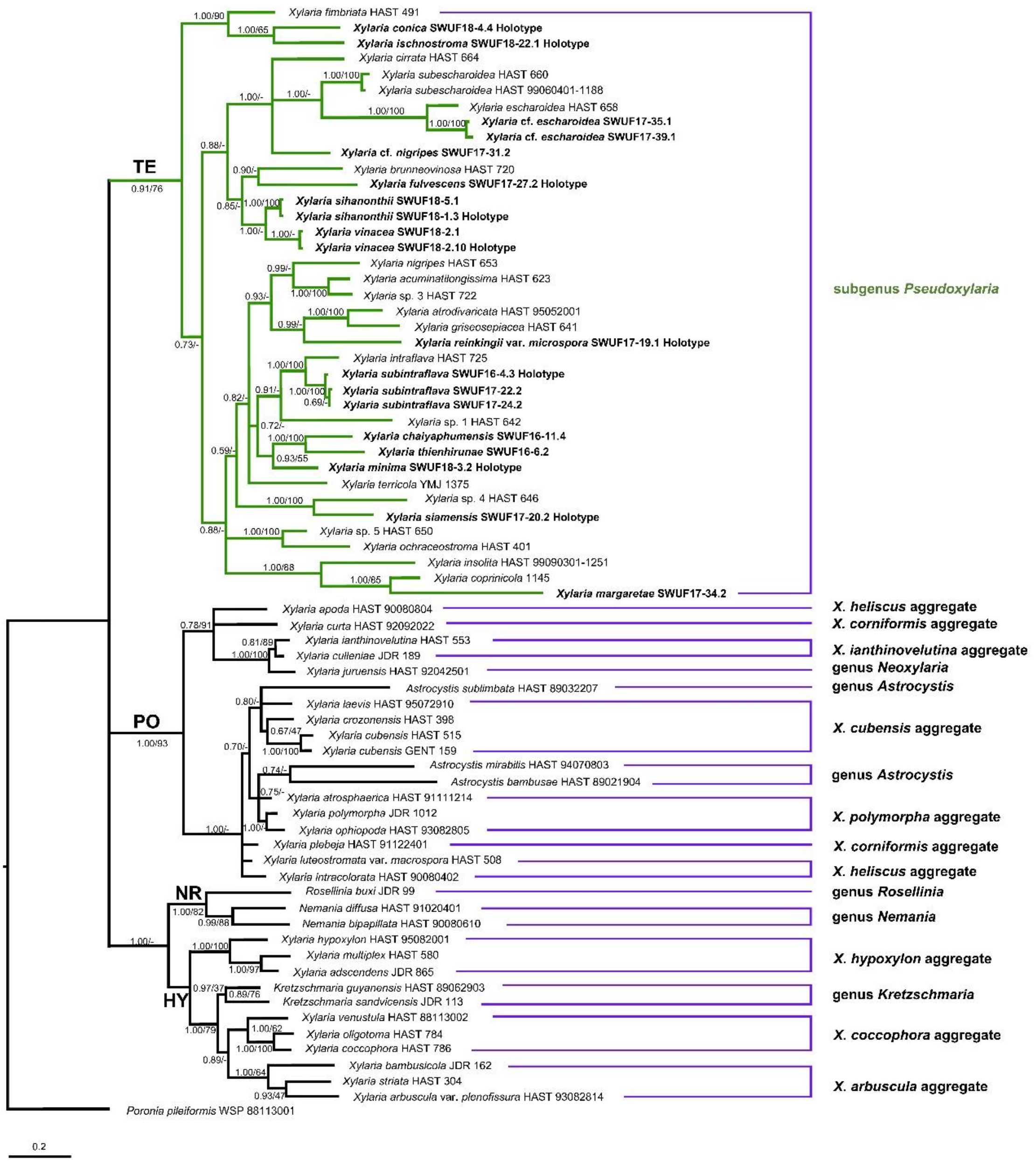

Figure S3. Phylogenetic tree inferred from BI analysis based on a dataset of intron ACT-TUB sequences of *Xylaria* species and related genera. Branches are labelled with Bayesian posterior probabilities and bootstrap values from BI and ML analyses, respectively. *Poronia pileiformis* (WSP 88113001) is the outgroup.

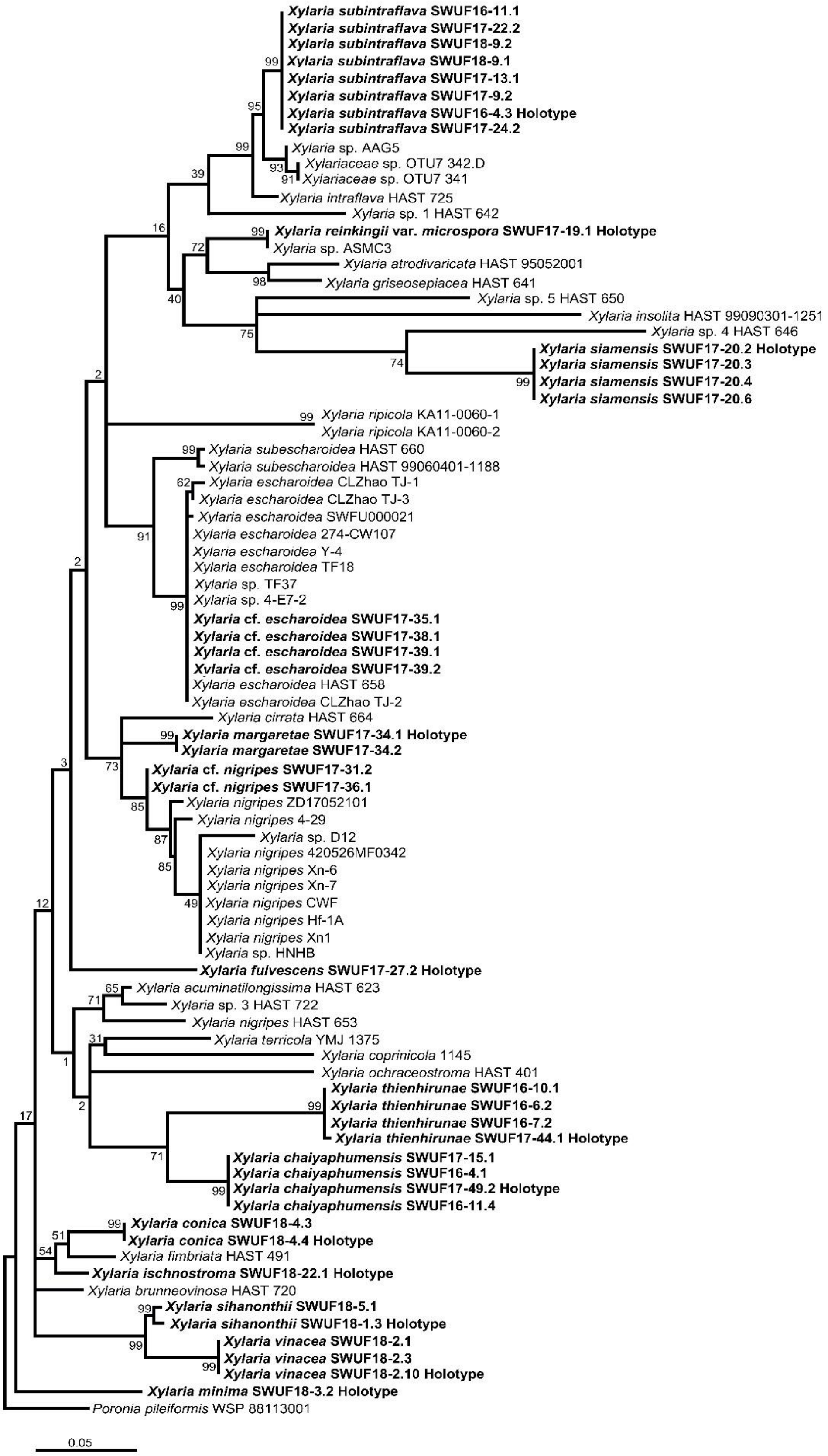

Figure S4. Phylogenetic tree inferred from ML analysis based on a dataset of ITS sequences of *Xylaria* species associated with termite nests. Branches are labelled with likelihood bootstrap support 1000 replications. *Poronia pileiformis* (WSP 88113001) is the outgroup.
